# The efficacy and safety of acupuncture therapy for sciatica: A systematic review and meta-analysis of randomized controlled trails

**DOI:** 10.3389/fnins.2023.1097830

**Published:** 2023-02-09

**Authors:** Zhihui Zhang, Tingting Hu, Peiyan Huang, Mengning Yang, Zheng Huang, Yawen Xia, Xinchang Zhang, Xiaolin Zhang, Guangxia Ni

**Affiliations:** ^1^College of Acupuncture-Moxibustion and Tuina, Nanjing University of Chinese Medicine, Nanjing, China; ^2^Key Laboratory of Acupuncture and Medicine Research of Ministry of Education, Nanjing University of Chinese Medicine, Nanjing, China; ^3^Department of Acupuncture and Rehabilitation, Jiangsu Province Hospital of Chinese Medicine, Affiliated Hospital of Nanjing University of Chinese Medicine, Nanjing, China; ^4^No. 1 Clinical Medical College, Nanjing University of Chinese Medicine, Nanjing, China; ^5^Department of Neurology, Affiliated Hospital of Jiangsu University, Zhenjiang, China

**Keywords:** acupuncture, sciatica, nerve pain, meta-analysis, systematic review

## Abstract

**Background and objective:**

Sciatica is a common type of neuropathic pain disease which poses a huge financial burden to the patient. For patients with sciatica, acupuncture has been recommended as an effective method for pain relief, while there is currently a lack of sufficient evidence to support its efficacy and safety. In this review, we aimed to critically assess the published clinical evidence on the efficacy and safety of acupuncture therapy for treating sciatica.

**Methods:**

An extensive literature search strategy was established in seven databases from their inception to 31 March 2022. Two independent reviewers performed the literature search, identification, and screening. Data extraction was performed on studies that meet the inclusion criteria, and a further quality assessment was performed according to the Cochrane Handbook and Standards for Reporting Interventions in Clinical Trials of Acupuncture (STRICTA) recommendations. Summary Risk ratio (RR) and standardized mean differences (SMDs) with 95% confidence interval (CI) were calculated using the fixed-effects or the random-effects model. Heterogeneity in effect size across studies was explored using the subgroup analysis and the sensitivity analysis. The quality of evidence was estimated following the Grading of Recommendations, Assessment, Development and Evaluations (GRADE) approach.

**Results:**

A total of 30 randomized controlled trials (RCTs) involving 2,662 participants were included in the meta-analysis. The results of the integration of clinical outcomes showed that the clinical efficacy of acupuncture was superior to that of medicine treatment (MT) in improving the total effective rate (relative risk (RR) = 1.25, 95% confidence interval (CI) [1.21, 1.30]; moderate certainty of evidence), reducing the Visual Analog Scale (VAS) pain score (standardized mean difference (SMD) = −1.72, 95% CI [-2.61, −0.84]; very low certainty of evidence), increasing pain threshold (SMD = 2.07, 95% CI [1.38, 2.75]; very low certainty of evidence), and decreasing recurrence rate (RR = 0.27, 95% CI [0.13, 0.56]; low certainty of evidence). In addition, a few adverse events (RR = 0.38, 95% CI [0.19, 0.72]; moderate certainty of evidence) were reported during the intervention, which indicated that acupuncture was a safe treatment option.

**Conclusions:**

Acupuncture therapy is an effective and safe treatment for patients with sciatica, and it can be considered a suitable replacement for medicine treatment (MT). However, given the high heterogeneity and a low methodological quality of previous studies, future RCTs should be well-designed according to the rigorous methodology.

**Systematic review registration:**

International Platform of Registered Systematic Review and Meta-analysis Protocols (INPLASY) (https://inplasy.com/register/), identifier [INPLASY202240060].

## Introduction

Sciatica, a common type of neuropathic pain, is characterized by radicular pain radiating from the lower back region and down to the leg, sometimes with or without numbness, paresthesia, and muscle weakness (Valat et al., 2010). These symptoms are mostly related to the compression of the spinal nerve root by disc herniation, accounting for 85% of the total cases (Ropper and Zafonte, 2015). The prevalence of sciatica varies widely from 1.2 to 43% with an annual incidence of 1–5% and a peak incidence in the fourth decade of life (Konstantinou et al., 2015; Davis et al., 2022). Sciatica is normally self-limiting with the relieving of pain over time in some cases (Oosterhuis et al., 2019). However, it remains more prevalent among certain populations, especially physical laborers (Fairag et al., 2022). Owing to the lack of an effective treatment, a significant proportion of patients with sciatica experience pain that persists for 1 year or longer. Persistent or unresolved pain could eventually lead to neurological deficits and functional disability, which have a serious impact on the quality of life (QoL) and pose a significant burden on the patient's healthcare resources (Maslak et al., 2020).

Seeking an appropriate method of treatment for sciatica is essential. Currently, the treatment options for sciatica can be classified into two categories: surgery and non-surgery, mainly depending on the severity of the condition. Patients with acute radicular pain may be considered for surgery, owing to the advantages of fast pain relief (Schoenfeld and Kang, 2020). However, the long-term efficacy of surgery remains to be determined. A systematic review reported that there were no differences in any clinical outcome (e.g., pain intensity, recurrence rate, and so on) between surgery and conservative care at 1- and 2-year follow-ups (Jacobs et al., 2011). Thus, the preferred treatment for the management of patients with sciatica is conservative, which includes exercise and manual therapy, medication, and spinal injections (Valat et al., 2010; Jensen et al., 2019). Based on the primary purpose of pain relief, analgesic drugs such as for example, non-steroidal anti-inflammatory drugs (NSAIDs) (Friedman et al., 2019) are often prescribed for patients with sciatica. However, several issues could arise from the use of NSAIDs, among which safety and adverse events are the most critical issues (Enthoven et al., 2016). As a result, it is imperative to search for effective and safe alternative pharmaceutical approaches.

Acupuncture therapy, as a non-pharmacological treatment derived from traditional Chinese medicine (TCM), is an established analgesic modality for treating pain. Modern medical research indicates that acupuncture exerts analgesic effects by regualting the activation of microglia, inhibiting inflammatory response and modulating certain receptors along the pain pathways in the central or peripheral nervous systems (Coutaux, 2017; Wang et al., 2020). The results of clinical studies on acupuncture therapy for sciatica showed that acupuncture is effective in relieving its symptoms (Liu et al., 2019; Yu et al., 2022). The effects of acupuncture treatment are also determined by selecting the appropriate acupuncture method, including manual acupuncture (MA) with twirling, lifting, and thrusting manipulation, electroacupuncture (EA) with an electric microcurrent device, and warm acupuncture (WA) with a combination of acupuncture and moxibustion treatment (Cao et al., 2021). According to the efficacy and safety of acupuncture for pain relief, patients with sciatica often give consent to undergo acupuncture treatment in China.

Two systematic reviews were performed to investigate the effectiveness of acupuncture for sciatica in 2015 (Ji et al., 2015; Qin et al., 2015); however, recent guidelines did not recommend acupuncture as a suitable treatment for sciatica, which mainly resulted from a limited sample size and a high interstudy heterogeneity (Jensen et al., 2019). In recent years, more randomized controlled trials (RCTs) have been published, and we plan to renew the included literature and conduct a comprehensive meta-analysis to evaluate the efficacy and safety of acupuncture therapy for sciatica.

## Methods

The protocol of this study was registered on the International Platform of Registered Systematic Review and Meta-analysis Protocols (INPLASY) (https://inplasy.com/register/), and the registration number was INPLASY202240060. This review was conducted according to the Preferred Reporting Items for Systematic Reviews and Meta-Analyses (PRISMA) (Page et al., 2021) (Supplementary Table 1).

### Literature search strategy

The databases, including three Chinese databases [China National Knowledge Infrastructure (CNKI), VIP Database for Chinese Technical Periodicals (VIP), and Wanfang Database] and four English databases (PubMed, Cochrane Library, Embase, and Web of Science), were searched for literature from their inception date until 31 March 2022. The key search terms were composed of the following group terms: (1) sciatica (sciatic neuralgia, sciatic pain, and sciatic neuropathy), (2) acupuncture (electroacupuncture, needle, needling, acupuncture and moxibustion, and warm acupuncture), and (3) sciatica plus acupuncture. The detailed search strategies for each database are presented in Supplementary Table 2.

### Inclusion criteria

The studies which were included must meet the following eligibility criteria.

#### Types of studies

In the design of studies, we included all RCTs which were used to evaluate the effectiveness and safety of acupuncture treatment for sciatica with no limitations set in language, blinding, or publication type.

#### Types of participants

Patients diagnosed with sciatica were included in this meta-analysis. The diagnostic criteria were based on symptoms, physical examination, medical imaging, and relevant published guidelines. There was no restriction in either age, gender, race, or ethnicity.

#### Types of interventions

The intervention of the experimental group was acupuncture therapy, including MA, EA, WA, and acupuncture plus moxibustion, regardless of acupoints, needle types, and materials. While in the control group (CG), the intervention was medicine treatment (MT), including conventional Western medicine or Chinese patent medicine. In addition, considering the potential placebo effects of acupuncture, sham acupuncture (SA) was included as another control intervention.

#### Types of outcome measures

The primary outcomes included total effective rate and pain intensity. The total effective rate was calculated by dividing the number of cured, markedly improved, and improved patients by the number of total patients. The pain intensity was measured by the Visual Analog Scale (VAS) with a 10-cm scale (0 cm represented no pain and 10 cm represented extreme pain). The secondary outcomes included the pain threshold, recurrence rate, and adverse events.

### Exclusion criteria

Studies were excluded if they did not meet the aforementioned criteria. In addition, the following studies were excluded if: (1) the types of studies included observational studies, animal studies, theoretical studies, data mining studies, thesis or dissertation, review, and meta-analyses; (2) the types of acupuncture included acupoint injections, laser acupuncture, cupping, and percutaneous stimulation; (3) interventions included a combination of acupuncture and medication; (4) the articles were duplicates; and (5) missing source literature or original data cannot be retrieved from the literature.

### Studies' selection and data extraction

The retrieved records were imported into NoteExpress, and the duplicates were removed. First, two reviewers (PY Huang and Z Huang) independently reviewed the titles and abstracts to eliminate irrelevant records and then read the full text to identify eligible studies. Finally, all relevant studies were retrieved for further assessment according to the inclusion and exclusion criteria. Disagreements were resolved through a team discussion and entrusted to a third reviewer (XC Zhang).

Two reviewers (ZH Zhang and TT Hu) independently extracted data from each included study by using a predesigned form. The general data of these studies were extracted, including the first author, publication year, sample size, diagnostic criteria, treatment details of treatment groups and control groups, outcome measures, follow-up period, and adverse events. After data extraction, each other's data were checked to ensure accuracy. When the results of the concerned study were ambiguous or incorrect, we contacted the authors for clarification and details. Meanwhile, we checked the source data to recalculate, and any disagreements were resolved *via* discussion with the third reviewer (XL Zhang).

### Quality assessment of risk of bias

Two reviewers (YW Xia and MN Yang) independently assessed the risk of bias for each included study. According to the Cochrane Handbook for Systematic Reviews of Interventions (version 5.1.0), the domains of bias included random sequence generation, allocation concealment, blinding method, incomplete outcome data, selective reporting, and other biases (Higgins and Green, 2011). For the risk of bias, “high risk of bias,” “low risk of bias,” or “unclear risk of bias” was assigned as the three levels to each domain. Any difference was resolved by discussion with a third reviewer (GX Ni) to reach a consensus.

### Quality assessment of acupuncture protocol

The detailed acupuncture treatment protocol of the included studies was assessed according to the Standards for Reporting Interventions in Clinical Trials of Acupuncture (STRICTA) checklist (MacPherson et al., 2010). The STRICTA checklist includes six items with 17 subitems, including the acupuncture rationale, details of needling, treatment regimen, other components of treatment, practitioner's background, and control or comparator interventions. We assessed the overall quality score (OQS) with 17 items from the STRICTA checklist (Zhuang et al., 2014). The score of each item was 0 or 1. If the item was completely reported, the score was “1,” but if the item was unreported or if the reported item was unclear, the score was “0.” In addition, the total score of each study was calculated to be in the range of 0 to 17, which indicated the rating of the overall reporting quality of an acupuncture protocol.

### Statistical analysis

Data analyses were conducted using RevMan (version 5.3) and R software (version 4.2.0). Continuous variables (i.e., pain threshold and pain intensity) were measured using the standardized mean difference (SMD) with a 95% confidence interval (CI), and dichotomous variables (i.e., total effective rate and recurrence rate) were measured using the risk ratio (RR) with a 95% CI. According to the Cochrane Handbook for Systematic Reviews of Interventions (Version 5.1.0), a value of *P* < 0.05 indicates a statistically significant difference (Higgins and Green, 2011). Cochrane's Q statistic and I^2^ statistic were used to inspect heterogeneity between studies. Heterogeneity was classified into two levels, when I^2^ is <50%, pooled effects of heterogeneous trials were calculated using the fixed-effects model, and when I^2^ is >50%, pooled effects of heterogeneous trials were calculated using the random-effects models.

#### Subgroup analysis

We performed the subgroup analysis based on the following aspects: (1) types of acupuncture interventions (i.e., MA, WA, and EA) and (2) sessions of acupuncture treatment (i.e., <15 or ≥15).

#### Sensitivity analysis

Sensitivity analysis was performed to verify the robustness of the results of the heterogeneity tests by eliminating studies case-by-case. In addition, the Baujat plot was used to further characterize the contribution to the overall heterogeneity in each study and identify high heterogeneity studies from the meta-analytic data (Baujat et al., 2002).

#### Publication bias

Publication bias was visually shown by funnel plots. In addition, we further formally tested the potential publication bias by using Egger's test or Peters' test, which are two significant testing methods based on the asymmetry of funnel plots. Egger's test was applied for continuous variables (i.e., pain intensity) and Peters' test was applied for dichotomous variables (i.e., total effective rate). If there was a value of *P* < 0.05, publication bias existed (Sterne et al., 2011).

#### Evidence quality assessment on GRADE

Based on the GRADE recommendations, we graded the quality of evidence through GRADEpro online software (https://www.gradepro.org/) (Atkins et al., 2004). The quality of included studies was graded high, moderate, low, or very low. The following aspects were used for assessment, including the risk of bias, inconsistent results, indirect evidence, imprecision, and publication bias.

## Results

### Search results

**Figure 1:**
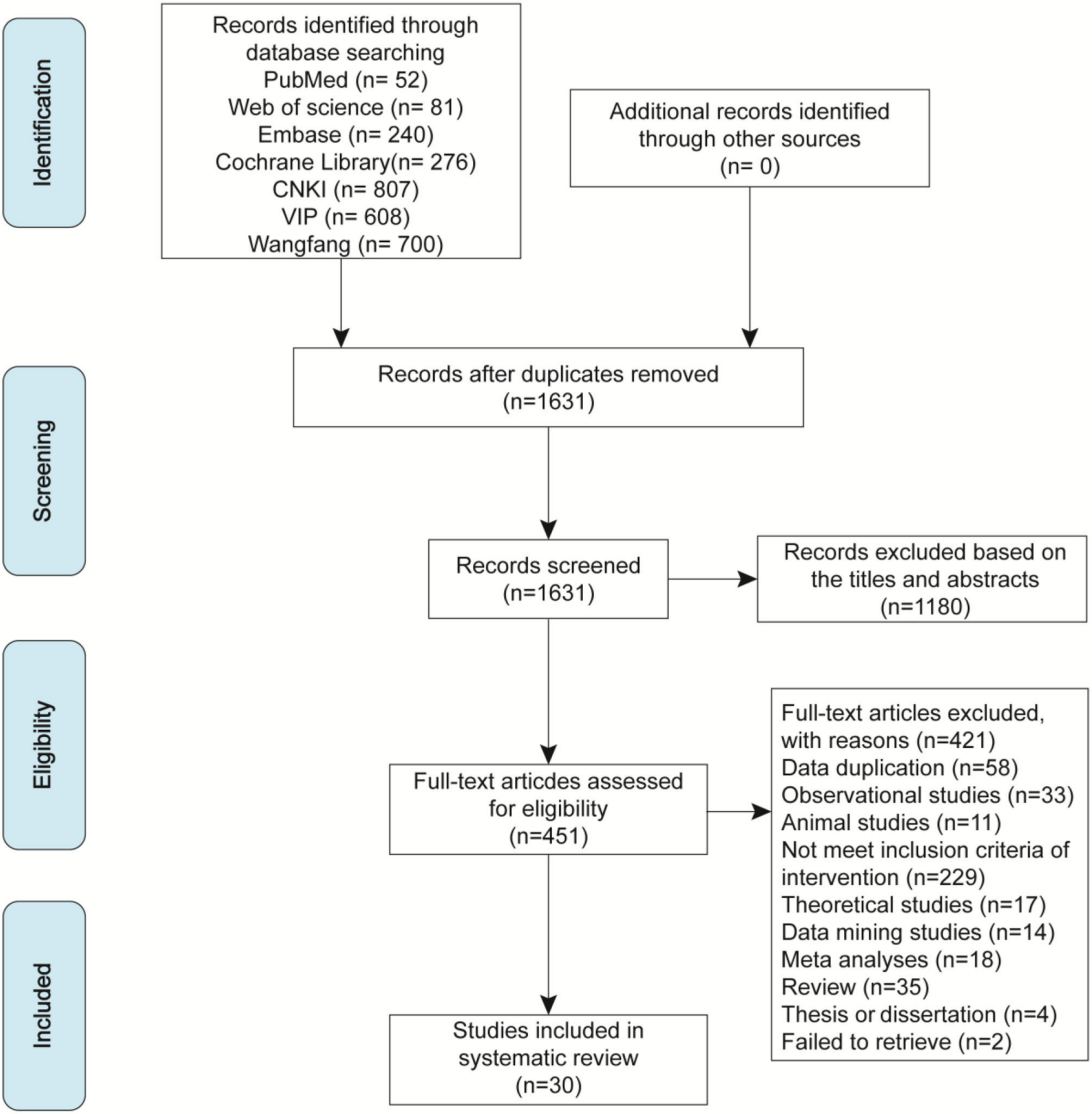
The Preferred Reporting Items for Systematic Reviews and Meta-Analyses (PRISMA) flowchart of the literature search and screening.

The flow diagram of the screening process is shown in Figure 1. The search retrieved 2,764 records. After duplicates were removed, 1,631 records were screened for potential relevance by reviewing titles and abstracts. Among these, 1,180 records were excluded, and the remaining 451 records required a full-text assessment. Through screening, we finally included 30 studies (Chen et al., 2005; Li and Meng, 2011; Jiang, 2012, 2018; Liu, 2012, 2015, 2017; Zeng and Liao, 2012; Zhai, 2012; Zhang, 2012; Shang et al., 2014; Ai, 2015; Huang et al., 2015, 2019; Nie, 2015; Ye et al., 2015; Li and Kang, 2016; Wang, 2016, 2017, 2020; Wei, 2016; Hu, 2017; Yu, 2017; Zou, 2017; Li, 2018; Zheng, 2019; Gu, 2020; Huo, 2020; Li et al., 2021). A total of 421 studies were excluded after screening the full text. The main reason for exclusion was that the intervention of studies did not meet the inclusion criteria. Additionally, we excluded animal studies, reviews, meta-analyses, theoretical studies, data mining studies, and theses or dissertations, among others because they were not randomized controlled trials (RCTs).

### Study characteristics

This review included 30 studies with 2,662 participants in total. Almost all studies were conducted in China, whereas two articles (Huang et al., 2019; Li et al., 2021) were English publications. The sample sizes ranged from 30 to 310 participants per study. The gender information on participants showed that the male-to-female ratio of the acupuncture group (AG) was 1.36 (575/423) and the male-to-female ratio of the control group was 1.43 (573/402), which were identified from 22 studies (Chen et al., 2005; Zhai, 2012; Shang et al., 2014; Ai, 2015; Huang et al., 2015, 2019; Liu, 2015, 2017; Ye et al., 2015; Li and Kang, 2016; Wang, 2016, 2017, 2020; Wei, 2016; Hu, 2017; Zou, 2017; Jiang, 2018; Li, 2018; Li et al., 2019, 2021; Zheng, 2019; Huo, 2020). All participants in the included trials must be diagnosed with sciatica. As regards diagnostic criteria, 10 studies used the published clinical guidelines as diagnostic criteria (Chen et al., 2005; Li and Meng, 2011; Liu, 2012; Zeng and Liao, 2012; Ai, 2015; Huang et al., 2015, 2019; Ye et al., 2015; Li et al., 2019, 2021), six studies reported the cause of sciatica induced by lumbar disc herniation (LDH) (Shang et al., 2014; Liu, 2015; Jiang, 2018; Zheng, 2019; Gu, 2020; Huo, 2020), seven studies were based on medical imaging as well as diagnostic criteria (Zhang, 2012; Liu, 2015; Wang, 2017; Yu, 2017; Li, 2018; Li et al., 2019; Gu, 2020), two studies used physical tests (i.e., straight-leg-raising test) (Li and Kang, 2016; Li et al., 2019), and nine studies did not report the diagnostic criteria (Jiang, 2012; Zhai, 2012; Nie, 2015; Wang, 2016, 2020; Wei, 2016; Hu, 2017; Liu, 2017; Zou, 2017). Acupuncture, MT, or SA was involved in intervention comparisons in studies. Twenty-eight studies compared acupuncture with MT (Chen et al., 2005; Li and Meng, 2011; Jiang, 2012, 2018; Liu, 2012, 2015, 2017; Zeng and Liao, 2012; Zhai, 2012; Zhang, 2012; Shang et al., 2014; Ai, 2015; Huang et al., 2015; Nie, 2015; Ye et al., 2015; Li and Kang, 2016; Wang, 2016, 2017, 2020; Wei, 2016; Hu, 2017; Yu, 2017; Zou, 2017; Li, 2018; Li et al., 2019; Zheng, 2019; Gu, 2020; Huo, 2020) and two studies compared acupuncture with SA (Huang et al., 2019; Li et al., 2021). Further details of these studies are summarized in Table 1.

**Table 1:** Characteristics of included studies.

**Included trails**	**Sample size (M/F)**		**Interventions**		**Diagnosis**	**Outcomes**	**Adverse events**	**Follow-up**
	**AG**	**CG**	**AG**	**CG (medicine/dosage/frequency)**				
Huo (2020)	60 (34/26)	60 (36/24)	Acupuncture	MT: Diclofanac Sodium Sustained Release Tablets/75 mg/qd	Sciatica caused by LDH	① ②	/	/
Gu (2020)	35	35	Acupuncture	MT: Brufen, Prednisone/NA/ NA	Sciatica caused by LDH/ medical imaging (MRI)	① ②	/	/
Zheng (2019)	155 (88/67)	155 (87/68)	Acupuncture	MT: Brufen/0.6 g/bid. Prednisone/10 mg/bid	Sciatica caused by LDH	①	/	/
Li et al. (2019)	46 (29/17)	46 (26/20)	Acupuncture	MT: Compound Mannitol Injection/125–250 ml/ NA. Dexamethasone/5–10 mg/ NA Aceclofenac Dispersible Tablets (1st week po./0.1 g/bid 2st week po./0.1 g/qd)	Physical tests/medical imaging (MRI/CT)/≪The clinical diagnostic and curative criteria of disease≫	① ②	/	/
Jiang (2018)	60 (38/22)	60 (37/23)	Acupuncture	MT: Brufen/0.6 g/tid Prednisone/10 mg/tid	Sciatica caused by LDH		Y	/
Zou (2017)	30 (17/13)	30 (16/14)	Acupuncture	MT: Nimesulide/0.1 g/ bid	NA	① ③	/	/
Yu (2017)	28	22	Acupuncture	MT: Indomethacin/30mg/bid Vitamin B12/500μg/qd	Medical imaging (CT/X-rays)	①	/	/
Liu (2015)	48 (29/19)	48 (30/18)	Acupuncture	MT: Brufen/0.6 g/d/tid Prednisone/10 mg/d/tid	Medical imaging (CT/MRI)/ sciatica caused by LDH	①	/	/
Shang et al. (2014)	60 (36/24)	60 (38/22)	Acupuncture	MT: Brufen/0.6 g/tid Prednisone/10 mg/tid	Sciatica caused by LDH	①	/	/
Liu (2017)	42 (28/14)	41 (27/14)	Acupuncture	MT: Nimesulide/0.1 g/bid	NA	① ②	/	/
Zeng and Liao (2012)	65	65	Acupuncture and moxibustion	MT: Brufen/0.6 g/tid Prednisone/10 mg/tid	Criteria of therapeutic effect and diagnosis of diseases and syndromes in TCM	①	/	/
Zhang (2012)	70	75	Acupuncture and moxibustion	MT: Brufen/0.6 g/tid Prednisone/10 mg/tid	Medical imaging (CT/X-rays)	①	/	/
Wang (2020)	40 (23/17)	40 (20/20)	Acupuncture and moxibustion	MT: Nimesulide/0.1 g/bid	NA	①	Y	/
Li (2018)	33 (24/9)	33 (25/8)	Acupuncture and moxibustion	MT: Brufen/0.6 g/tid Prednisone/10 mg/tid	medical imaging (CT/X-rays)	①	/	/
Wang (2016)	90 (51/39)	90 (48/32)	Acupuncture and moxibustion	MT: Brufen/0.6 g/tid Prednisone/10 mg/tid	NA	①	/	/
Wang (2017)	25 (14/11)	25 (15/10)	Acupuncture and moxibustion	MT: Brufen/0.6 g/tid Prednisone/10 mg/tid	medical imaging (CT/X-rays)	①	/	/
Nie (2015)	39	37	Acupuncture and moxibustion	MT: Nimesulide/0.1 g/bid	NA	①	/	/
Jiang (2012)	41	41	Acupuncture and moxibustion	MT: Brufen/0.6 g/tid Prednisone/10 mg/tid	NA	①	/	/
Liu (2012)	30	20	Acupuncture and moxibustion	MT: Nimesulide/0.1 g/bid	≪The clinical diagnostic and curative criteria of disease≫	③	/	/
Li and Kang (2016)	30 (19/11)	30 (22/8)	Acupuncture and moxibustion	MT	Physical tests	①	/	/
Hu (2017)	40 (20/20)	40 (21/19)	Warm acupuncture	MT: Nimesulide/0.1 g/bid	NA	① ④	Y	Y
Wei (2016)	15 (12/3)	15 (9/6)	Warm acupuncture	MT: Nimesulide/0.2 g/bid	NA	① ④	/	Y
Ai (2015)	30 (20/10)	30 (21/9)	Warm acupuncture	MT: Nimesulide/0.3 g/bid	The clinical diagnostic and curative criteria of disease/Criteria of therapeutic effect and diagnosis of diseases and syndromes in TCM	①	/	/
Zhai (2012)	28 (17/11)	28 (16/12)	Warm Acupuncture	MT: Nimesulide/0.4 g/bid	NA	①	/	/
Chen et al. (2005)	30 (22/8)	30 (21/9)	Warm Acupuncture	MT: Nimesulide/0.5 g/bid	The clinical diagnostic and curative criteria of disease (1999)	① ③	/	/
Huang et al. (2015)	35 (24/11)	35 (27/8)	Electroacupuncture	MT: Brufen/0.3 g/bid Mecobalamin/0.5 mg/tid	Criteria of therapeutic effect and diagnosis of diseases and syndromes in TCM	① ②	/	/
Li and Meng (2011)	49	37	Electroacupuncture	MT: Brufen/0.3 g/bid VitaminB1/20 mg/tid	3,200 standard diagnoses of diseases in internal medicine	① ② ④	/	Y
Ye et al. (2015)	31 (12/19)	30 (11/19)	Electroacupuncture	MT: Dichofenac Diethylammon (drug external)/NA/ qid	Criteria of therapeutic effect and diagnosis of diseases and syndromes in TCM/Guiding principle of clinical research on new drugs of TCM (trial)	②	/	/
Huang et al. (2019)	23 (7/16)	23 (8/15)	Acupuncture	SA	The North American Spine Society clinical guidelines	②	Y	Y
Li et al. (2021)	37 (11/26)	36 (12/24)	Acupuncture	SA	Inclusion criteria-Patients with unilateral sciatica who meet the diagnostic criteria(P6)	②	Y	Y

①total effective rate; ②pain intensity; ③pain threshold; ④recurrence rate.

AG, acupuncture group; CG, control group; NA, Not applicable; MT, medicine treatment; SA, sham acupuncture; LDH, lumbar disc herniation.

### Acupuncture therapy protocols of included trials

A total of 27 studies used MA (Chen et al., 2005; Jiang, 2012, 2018; Liu, 2012, 2015, 2017; Zeng and Liao, 2012; Zhai, 2012; Zhang, 2012; Shang et al., 2014; Ai, 2015; Nie, 2015; Li and Kang, 2016; Wang, 2016, 2017, 2020; Wei, 2016; Hu, 2017; Yu, 2017; Zou, 2017; Li, 2018; Huang et al., 2019; Li et al., 2019, 2021; Zheng, 2019; Gu, 2020; Huo, 2020) and the rest of the three studies used EA (Li and Meng, 2011; Huang et al., 2015; Ye et al., 2015). As for the intervention types of MA, 12 studies used only needles (Shang et al., 2014; Liu, 2015, 2017; Yu, 2017; Zou, 2017; Jiang, 2018; Huang et al., 2019; Li et al., 2019, 2021; Zheng, 2019; Gu, 2020; Huo, 2020), 10 studies used acupuncture and moxibustion (Jiang, 2012; Liu, 2012; Zeng and Liao, 2012; Zhang, 2012; Nie, 2015; Li and Kang, 2016; Wang, 2016, 2017, 2020; Li, 2018), and five studies used WA (Chen et al., 2005; Zhai, 2012; Ai, 2015; Wei, 2016; Hu, 2017). All included studies reported the choice of acupoints. As shown in **Figure 3A**, the most frequent acupoints were GB30, BL25, BL4, BL60, BL23, BL54, and GB34. A total of 26 studies reported the retention time of needles (Chen et al., 2005; Li and Meng, 2011; Jiang, 2012, 2018; Liu, 2012, 2017; Zeng and Liao, 2012; Zhai, 2012; Zhang, 2012; Shang et al., 2014; Ai, 2015; Huang et al., 2015, 2019; Nie, 2015; Ye et al., 2015; Li and Kang, 2016; Wang, 2016, 2017, 2020; Wei, 2016; Hu, 2017; Yu, 2017; Li, 2018; Li et al., 2019, 2021; Huo, 2020). It was reported that the retention time ranged mostly from 15 to 30 min. Only two studies showed that the retention time was only 5 min (Liu, 2012; Nie, 2015). The frequency of treatment was one time a day (Jiang, 2012, 2018; Liu, 2012, 2017; Zeng and Liao, 2012; Zhang, 2012; Shang et al., 2014; Nie, 2015; Li and Kang, 2016; Wang, 2016, 2017, 2020; Wei, 2016; Hu, 2017; Yu, 2017; Zou, 2017; Li, 2018; Li et al., 2019; Huo, 2020) and one time every other day (Huang et al., 2019). Details of the acupuncture intervention are summarized in Table 2.

**Table 2:** Details of characteristics of acupuncture intervention.

**Included trails**	**Acupuncture Style**	**Acupoints formula**	**Details of acupuncture therapy**		**Treatment regimen**			**Other acupuncture treatment**
			**Needle stimulation**	**Needle type**	**Retention time**	**Session**	**Frequency**	
Huo (2020)	TCM	BL57, BL25, GB30, BL40, BL54, GB34, GB30, GB39, ABL60, BL25, GB30, GB34	Manual	(1) GB30, BL54: 0.30 × 75 mm; (2) other acupoints: 0.25 × 40 mm	30 min	15	once a day	/
Gu (2020)	TCM	Pain syndromes or near the site of pain	Manual	According to site of acupoints	NA	NA	/	/
Zheng (2019)	TCM	GB30, BL40, BL57, BL25, GB34, GB30, GB39	Manual	/	NA	NA	/	/
Li et al. (2019)	TCM	GB30, BL54, GB39, GB34, GB30, BL57, BL40, BL25, GB30, BL60, GB34, BL25	Manual	(1) GB30, BL54: 0.30 × 75 mm; (2) other acupoints: 0.25 × 40 mm	30 min	14	once a day	/
Jiang (2018)	TCM	GB30, GB39, GB34, BL25, GB30, BL57, BL40, GB30, BL25, GB34, BL60	Manual	/	30 min	14	once a day	/
Zou (2017)	TCM	BL23, GB30, BL25, BL60, BL40	Manual	/	NA	23	once a day	/
Yu (2017)	TCM	BL57, GB30, BL25, BL40, GB30, ST40, GB34, GB39, BL25, BL54, GB34, GB30, BL60	Manual	0.45 × 150 mm	30 min	14	once a day	/
Liu (2015)	TCM	GB30, BL25, BL40, BL57, GB30, GB34, GB39, BL25, GB30, GB34, BL60.	Manual	According to the site of acupoints	NA	NA	/	/
Shang et al. (2014)	TCM	GB30, BL25, BL40, BL57, GB30, GB34, GB39.	Manual	0.45 × 150 mm; 0.45 × 40–75 mm	30 min	14	once a day	/
Liu (2017)	TCM	BL23, GB30, BL40, BL57, BL25, BL60, GB34, BL54, BL23, GB30, GB34, BL25, GB40, GB39, SP9, LR2, LI11, SP10, BL26, ST36, SP10, BL18, BL17	Manual	/	30 min	23	once a day	/
Zeng and Liao (2012)	TCM	BL25, GB30, BL54, GB34, BL60	Manual	/	30 min	14	once a day	Indirect Moxibustion
Zhang (2012)	TCM	BL25, GB30, BL40, BL57, GB30, GB34, ST40, GB39, BL25, GB30, BL54, GB34, BL60	Manual	/	30 min	14	once a day	Indirect Moxibustion
Wang (2020)	TCM	BL25, BL23, GB30, BL40, BL60	Manual	0.3 × 60–75 mm	25 min	10	once a day	Direct Moxibustion
Li (2018)	TCM	GB34, BL40, BL54, GB30, EX-B2, BL25, GV3, BL23, ST32, GB31, BL36, BL26, BL60, GB39, GB34, BL57, BL32	Manual	/	30 min	23	once a day	Indirect Moxibustion
Wang (2016)	TCM	GB30, BL25, BL54, BL60, BL23, BL40, GB34	Manual	0.3 × 70 mm	20–30 min	21	once a day	Indirect Moxibustion
Wang (2017)	TCM	BL40, GB30, BL57, BL25, GB39, ST40, GB30, GB34, BL60, GB30, BL54, BL25, GB34, BL20, SP9, BL23, GV3, BL40,BL32, BL17, ST36, SP6	Manual	0.45 × 150 mm	30 min	29	once a day	Indirect Moxibustion
Nie (2015)	TCM	BL23, GB30, BL25, BL60, BL40	Manual	0.3 × 60–75 mm	5 min	23	once a day	Direct Moxibustion
Jiang (2012)	TCM	GB30, BL25, BL54, BL60, GB34	Manual	/	30 min	28	once a day	Indirect Moxibustion
Liu (2012)	TCM	BL23, BL25, GB30, BL40, BL60	Manual	0.3 × 60–75 mm	5 min	23	once a day	Direct Moxibustion
Li and Kang (2016)	TCM	BL32, GB30, BL54, BL40	Manual	/	30 min	15	once a day	Heat-Sensitive Moxibustion
Hu (2017)	TCM	BL23, BL25, GB30, BL54, ST33, BL40, BL60	Manual	(1) BL23, BL25, BL40: 0.30 × 40 mm; (2) GB30, BL54, ST33:0.30 × 60 mm; (3) BL60: 0.25 × 30 mm	30 min	10	once a day	Warm Acupuncture
Wei (2016)	TCM	BL23, BL60, BL40, GB30, BL25	Manual	0.3 × 60-75 mm	25min	23	once a day	Warm Acupuncture
Ai (2015)	TCM	BL23, BL25, GB30, BL40, BL60	Manual	0.3 × 65 mm	15–30 min	21	once a day	Warm Acupuncture
Zhai (2012)	TCM	BL23, BL25, GB30, BL40, BL60	Manual	0.3 × 60–75 mm	15–30 min	23	once a day	Warm Acupuncture
Chen et al. (2005)	TCM	BL23, BL25, GB30, BL40, BL60	Manual	0.3 × 60–75 mm	15–30 min	23	once a day	Warm Acupuncture
Huang et al. (2015)	TCM	BL25, BL26, GB30, BL40, BL54, BL36, ST32, GB31, GB39, BL60; BL57, BL32, GB30	Electrical	/	30 min	22	once a day	/
Li and Meng (2011)	TCM	GB30, GB34, BL57, BL60, BL54, BL40, GB31, GB39, GB41	Electrical	0.3 × 40 mm	30 min	27	once a day	/
Ye et al. (2015)	TCM	EX-B2 (L4-5, L5-S1), BL54, GB30	Electrical	/	30 min	21	once a day	/
Huang et al. (2019)	TCM	BL25, BL23, BL40, BL57	Manual	0.35 × 75 mm; 0.35 × 40 mm	30 min	28	once every other day	/
Li et al. (2021)	TCM	BL25	Manual	/	30 min	112	/	/

TCM, Traditional Chinese medicine; NA, not applicable.

### Risk of bias assessment

**Figure 2:**
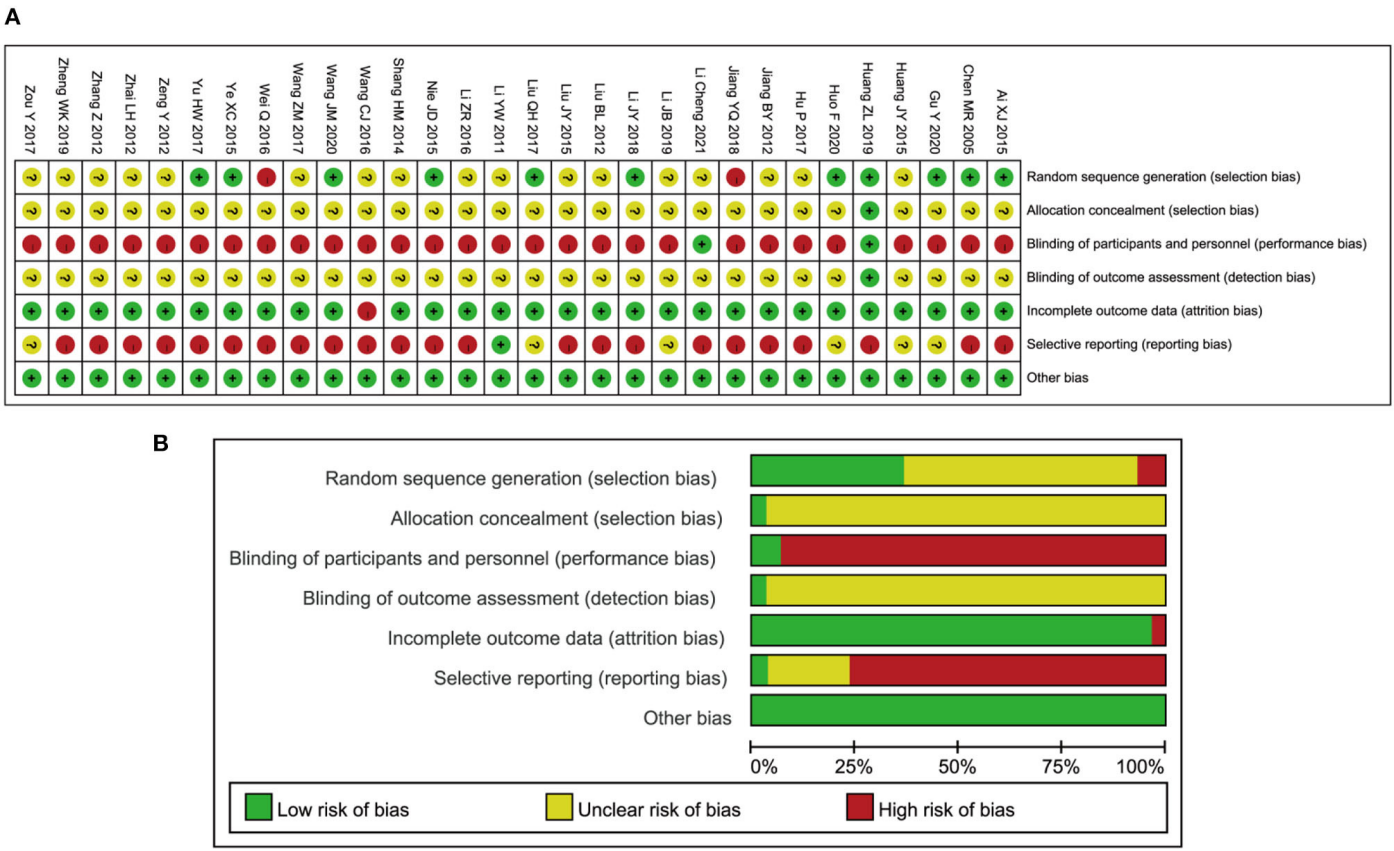
Risk of bias from included studies. **(A)** Risk of bias summary. **(B)** Risk of bias graph.

The results of the “risk of bias” assessment by domain for each study are displayed in Figure 2A, and the percentage results of risk evaluation in each domain are provided in Figure 2B. The specific reasons for the judgments are shown in Supplementary Table 3. In all studies, one to three domains were judged to be at high risk of bias. The main issue in most of the studies (28 studies, more than 90%) was the high risk of performance bias due to nonblinding of participants and personnel, which was related to the characteristic of acupuncture. During the process of acupuncture treatment, it is hard to implement blind procedures for acupuncturists and patients. Only two studies (Huang et al., 2019; Li et al., 2021) were judged to have a low risk of performance bias because they used acupuncture with sham intervention, which ensured that the participants were blinded. One study had a low risk of detection bias (Huang et al., 2019), while the rest of the studies had an unclear detection bias risk, as there was no indication of whether the assessors were blinded or not. A total of 11 studies mentioned random methods, including the random number table, randomized controlled parallel design, and computer-based random number generator (Chen et al., 2005; Ai, 2015; Nie, 2015; Ye et al., 2015; Liu, 2017; Yu, 2017; Li, 2018; Huang et al., 2019; Gu, 2020; Huo, 2020; Wang, 2020). However, two studies were judged to have a high selection bias because the sequence was generated by the time of admission (Wei, 2016; Jiang, 2018). The remaining studies were rated as “unclear risk” due to insufficient information to permit judgment of the sequence generation process (Li and Meng, 2011; Jiang, 2012; Liu, 2012, 2015; Zeng and Liao, 2012; Zhai, 2012; Zhang, 2012; Shang et al., 2014; Huang et al., 2015; Li and Kang, 2016; Wang, 2016, 2017; Hu, 2017; Zou, 2017; Li et al., 2019, 2021; Zheng, 2019). For the assessment of incomplete outcome data, almost all studies were graded to be at low risk of attrition bias. Among these, the data from three studies were found to contain mistakes which were later modified (Jiang, 2012; Zhang, 2012; Shang et al., 2014). Only one study was rated to be at high attrition bias risk because the data on pain threshold were not reported (Wang, 2016).

### STRICTA checklist for the included studies

**Figure 3:**
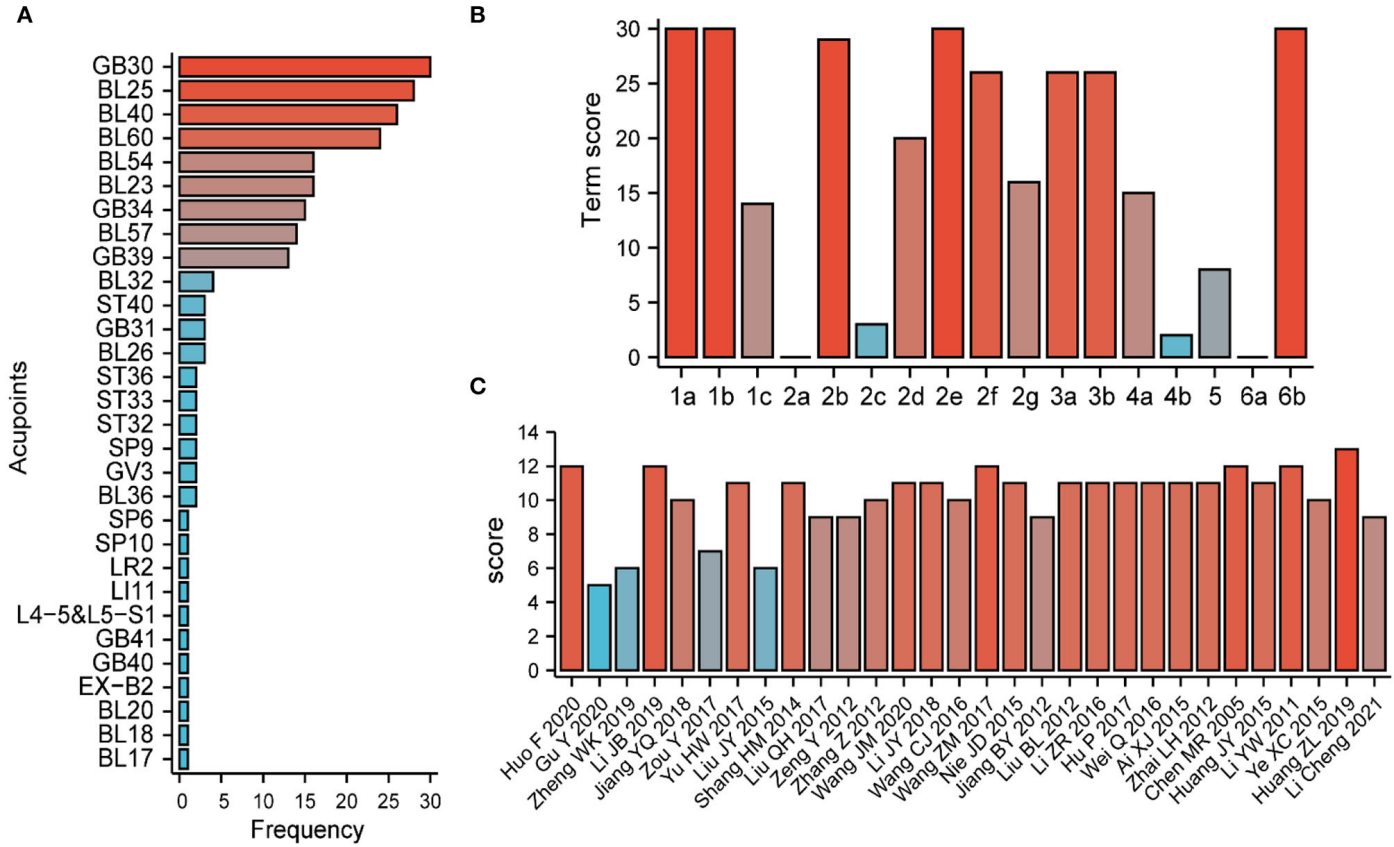
Acupuncture details and Standards for Reporting Interventions in Clinical Trials of Acupuncture (STRICTA) checklist summary. **(A)** The frequency of acupoints in the included studies. **(B)** The total score for each STRICTA term. **(C)** The total STRICTA checklist score for each included study.

The summary of the assessment report on acupuncture details is provided in Supplementary Table 4 using the STRICTA checklist. As shown in Figure 3B, almost all studies reported the style of acupuncture (1a), treatment reasoning (1b), acupoints (2b), needle stimulation (2e), and a precise description of the control group (6b); more than half of the studies mentioned the retention time (2f), the number of treatment sessions (3a), the frequency of treatment sessions (3b), the frequency of responses sought (2d), the needle type (2g), and the details of other interventions (4a); less than half of the studies reported the depth of insertion (2c), places and facilities of treatment (4b), and description of participating acupuncturists (5). The OQS from the STRICTA checklist of each study is presented in Figure 3C. The scores of 22 studies were ≥10 (Chen et al., 2005; Li and Meng, 2011; Liu, 2012; Zhai, 2012; Zhang, 2012; Shang et al., 2014; Ai, 2015; Huang et al., 2015, 2019; Nie, 2015; Ye et al., 2015; Li and Kang, 2016; Wang, 2016, 2017, 2020; Wei, 2016; Hu, 2017; Yu, 2017; Jiang, 2018; Li, 2018; Li et al., 2019; Huo, 2020), while the rest of the studies scored < 10 (Jiang, 2012; Zeng and Liao, 2012; Liu, 2015, 2017; Zou, 2017; Zheng, 2019; Gu, 2020; Li et al., 2021). The overall reporting quality of interventions in controlled trials of acupuncture was relatively good.

### Effects of interventions

#### Primary outcomes

##### Total effective rate

**Figure 4:**
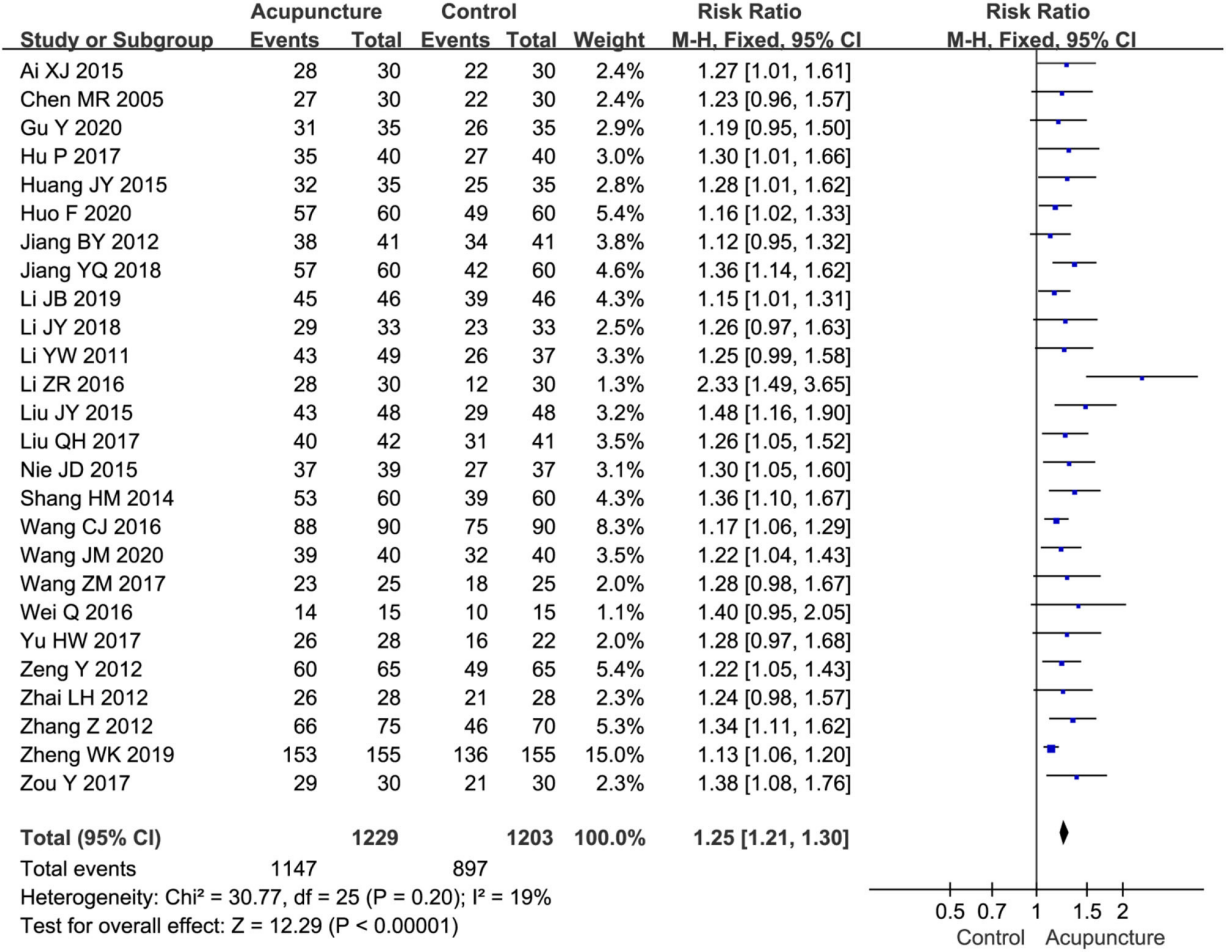
A Forest plot for total effective rate.

The total effective rate was reported in 26 studies (Chen et al., 2005; Li and Meng, 2011; Jiang, 2012, 2018; Zeng and Liao, 2012; Zhai, 2012; Zhang, 2012; Shang et al., 2014; Ai, 2015; Huang et al., 2015; Liu, 2015, 2017; Nie, 2015; Li and Kang, 2016; Wang, 2016, 2017, 2020; Wei, 2016; Hu, 2017; Yu, 2017; Zou, 2017; Li, 2018; Li et al., 2019; Zheng, 2019; Gu, 2020; Huo, 2020) in which the efficacy of acupuncture therapy was compared with that of MT on sciatica. The results of the meta-analysis revealed that the total effectiveness of acupuncture therapy was statistically significantly better than that of MT (RR = 1.25, 95%CI [1.21, 1.30], *P* < 0.00001) (Figure 4).

The results of subgroup analyses are summarized in Table 3. With regard to the types of acupuncture interventions, the results of subgroup analyses presented that MA (RR=1.25, 95%CI [1.21, 1.30]), WA (RR = 1.27, 95%CI [1.13, 1.43]), and EA (RR = 1.26, 95%CI [1.07, 1.49]) were superior to MT in improving the total effective rate. As for treatment sessions, we found that acupuncture treatments of <15 sessions (RR = 1.28, 95%CI [1.20, 1.37]) and ≥15 sessions (RR = 1.24, 95%CI [1.19, 1.29]) exhibited a statistically significant effect in improving the total effective rate compared with MT.

**Table 3:** The subgroup analysis for the outcomes of included studies.

**Subgroup**	**Eligible studies**	**Intervention group (n)**	**Control group (n)**	**RR/SMD (95% CI)**	***P*** **value**	**Heterogeneity test**	**Effect model**
*Total effective rate*							
Acupuncture categories							
MA vs. MT	19	1,002	988	1.25 [1.20, 1.30]	<0.001	*P =* 0.04, I^2^ = 39%	Fixed
WA vs. MT	5	143	143	1.27 [1.13, 1.43]	<0.001	*P =* 0.98, I^2^ = 0%	Fixed
EA vs. MT	2	84	72	1.26 [1.07, 1.49]	<0.001	*P =* 0.88, I^2^ = 0%	Fixed
Total sessions of treatment							
< 15	8	381	290	1.28 [1.20, 1.37]	<0.001	*P =* 0.77, I^2^ = 0%	Fixed
Greater than or equal to 15	17	723	581	1.24 [1.19, 1.29]	<0.001	*P =* 0.08, I^2^ = 35%	Fixed
*Pain intensity*							
Acupuncture categories							
MA vs. MT	4	183	182	−3.16 [−4.48, −1.83]	<0.001	P < 0.01, I^2^ = 94%	Random
EA vs. MT	3	115	102	−0.50 [−0.89, −0.12]	<0.001	*P =* 0.14, I^2^ = 48%	Fixed
RA vs. SA	2	60	59	−0.34 [−0.89, 0.20]	0.22	*P =* 0.14, I^2^ = 53%	Random
Total sessions of treatment							
< 15	3	100	99	−1.98 [−4.06, 0.10]	0.060	P < 0.01, I^2^ = 97%	Random
Greater than or equal to 15	4	186	173	−2.08 [−3.96, −0.19]	0.030	P < 0.01, I^2^ = 98%	Random
*Pain threshold*							
Acupuncture categories							
MA vs. MT	2	60	50	1.82 [1.06, 2.59]	<0.001	*P =* 0.10, I^2^ = 64%	Random
WA vs. MT	1	30	30	2.57 [1.88, 3.27]	<0.001	NA	Random
*Recurrence rate*							
Acupuncture categories							
WA vs. MT	2	55	55	0.38 [0.16, 0.88]	0.020	*P =* 0.90, I^2^ = 0%	Fixed
EA vs. MT	1	49	37	0.14 [0.03, 0.58]	0.007	NA	Random

RR, risk ratio; SMD, standardized mean difference; 95% CI, 95% confidence interval; MT, medicine treatment; MA, manual acupuncture; EA, electroacupuncture; WA, warm acupuncture; RA, real acupuncture; SA, sham acupuncture; NA, not applicable.

##### Pain intensity

**Figure 5:**
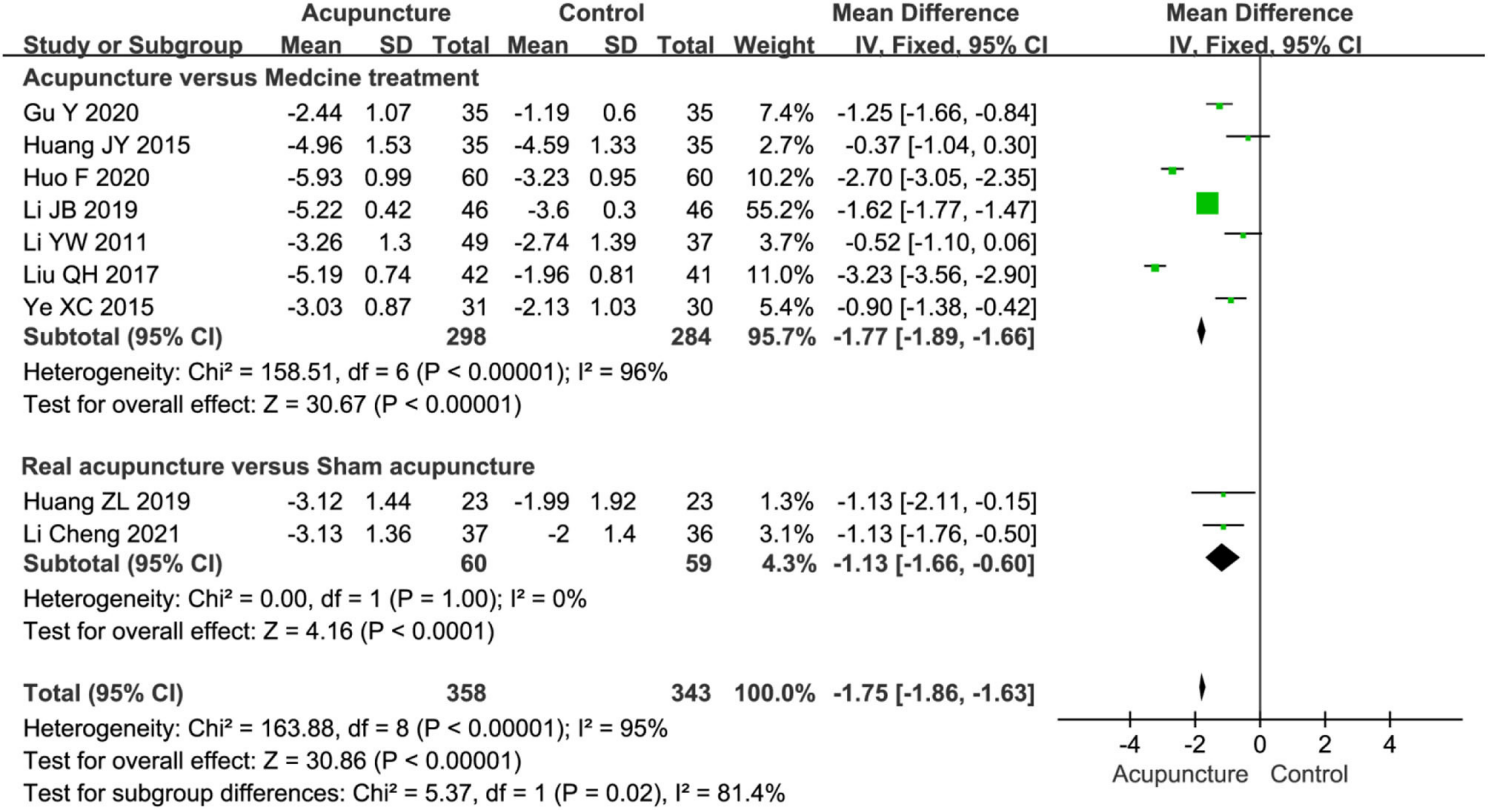
A Forest plot for pain intensity.

Nine studies, including 701 participants, used the VAS score (0–10cm scale) to calculate the pain intensity of acupuncture for sciatica. Among these, seven studies (Li and Meng, 2011; Huang et al., 2015; Ye et al., 2015; Liu, 2017; Li et al., 2019; Gu, 2020; Huo, 2020) compared acupuncture with MT, and two studies (Huang et al., 2019; Li et al., 2021) compared real acupuncture with SA. The results of the VAS score in the acupuncture group showed a statistically significantly lower value than that in the MT group (MD = −1.77, 95%CI [−1.89, −1.66], *P* < 0.00001) (Figure 5). In addition, two studies (Huang et al., 2019; Li et al., 2021) reported that real acupuncture was statistically superior to SA in improving the VAS score for sciatic pain (MD = −1.13, 95%CI [-1.66, −0.60], *P* < 0.0001) (Figure 5), and there was no evidence of heterogeneity (*P* = 1.00, I^2^ = 0%).

The subgroup analysis indicated that both MA (SMD = −3.16, 95%CI [−4.48, −1.83]) and EA (SMD=-0.50, 95%CI [−0.89, −0.12]) reduced the VAS score more than MT. However, there was high heterogeneity (I^2^ = 94%) in the comparison of MA vs. MT. For the sessions of acupuncture therapy, we found that acupuncture therapy with ≥15 sessions (SMD = −1.86, 95%CI [−3.50, −0.22]) had a better effect of reducing the VAS score than MT, while it had a little effect within 15 sessions (SMD = −1.98, 95%CI [−4.06, 0.10]) (Table 3).

#### Secondary outcomes

##### Pain threshold

Three studies with 170 participants examined the effects of acupuncture therapy on the pain threshold induced by sciatica vs. MT (Chen et al., 2005; Liu, 2012; Zou, 2017). The pooled results indicated that acupuncture had a statistically significantly better effect than medicine in improving pain threshold (SMD = 2.07, 95%CI [1.38, 2.75], *P* < 0.00001) (Supplementary Figure 1). The subgroup analysis showed that both MA (SMD = 1.82, 95%CI [1.06, 2.59]) and WA (SMD=2.57, 95%CI [1.88, 3.27]) were statistically significantly better than MT where the pain threshold increased (Table 3).

##### Recurrence rate

The data of recurrence rate during follow-up were obtained in three studies (Li and Meng, 2011; Wei, 2016; Hu, 2017). The pooled results showed that acupuncture had a superior long-term effect in reducing the occurrence of relapse for sciatic pain than MT (RR=0.27, 95%CI [0.13, 0.56]) (Supplementary Figure 2). There was no significant heterogeneity between the three studies (*P* = 0.49, I^2^ = 0%). The subgroup analysis indicated that WA (RR=0.38, 95%CI [0.16, 0.88]) and EA (RR=0.14, 95%CI [0.03, 0.58]) had a superior long-term effect in reducing the recurrence rate than MT (Table 3).

##### Adverse events

**Figure 6:**
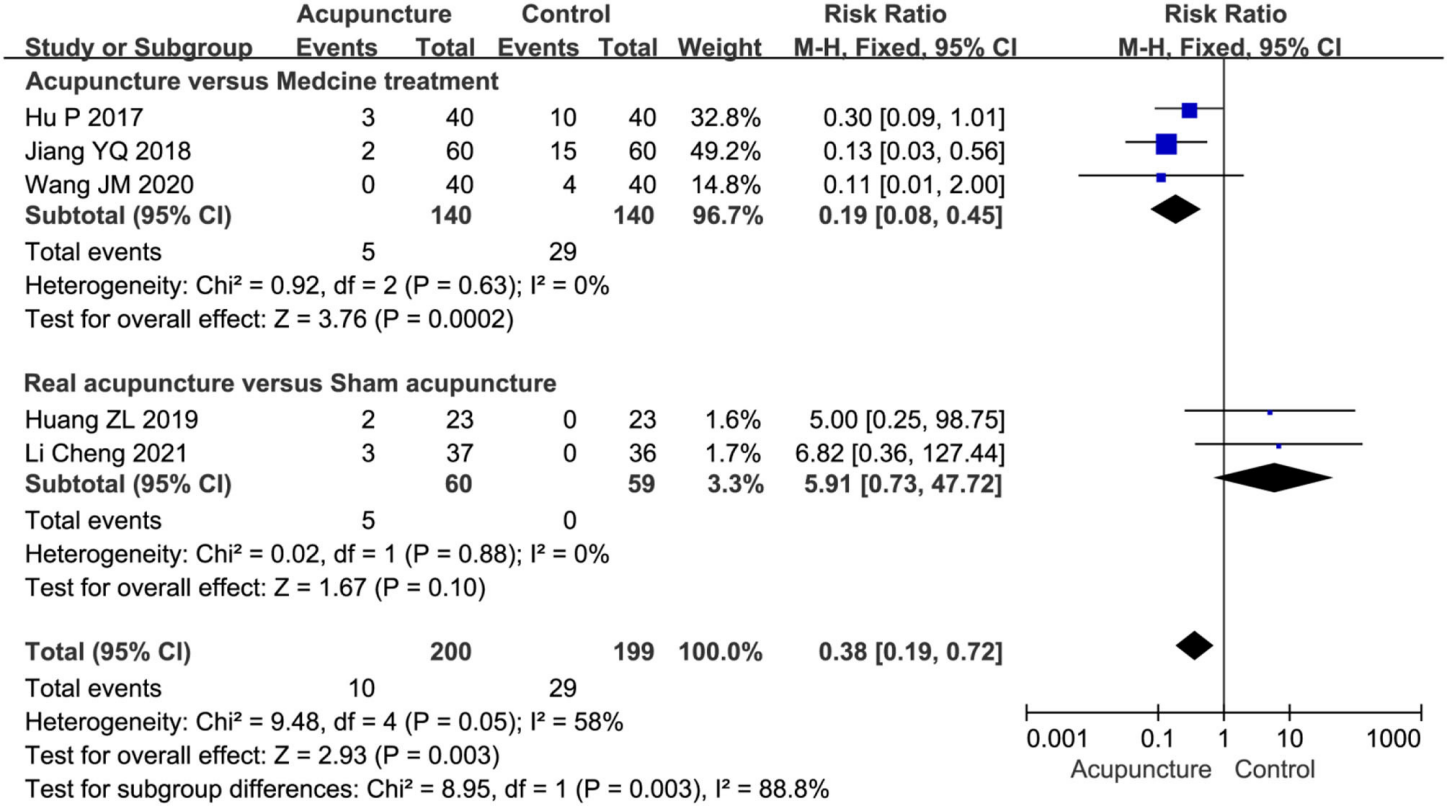
A Forest plot for adverse events.

Several adverse events took place during the treatment and were reported in five studies (Hu, 2017; Jiang, 2018; Huang et al., 2019; Wang, 2020; Li et al., 2021). We evaluated the incidence of adverse events by the subgroup analysis, including acupuncture vs. MT and real acupuncture vs. SA. The pooled results indicated a higher incidence rate of adverse effects in drug reactions compared with acupuncture (RR=0.19, 95%CI [0.08, 0.45]) (Figure 6). The adverse events of MT included dizziness, edema, gastrointestinal bleeding, acne, heart failure, and heartburn. Although subcutaneous hematoma and pinhole hemorrhage appeared occasionally in the process of acupuncture therapy, there was no statistically significant difference between real acupuncture and SA based on the two studies (Huang et al., 2019; Li et al., 2021) (RR = 5.91, 95%CI [0.73, 47.72]) (Figure 6).

### Sensitivity analysis

**Figure 7:**
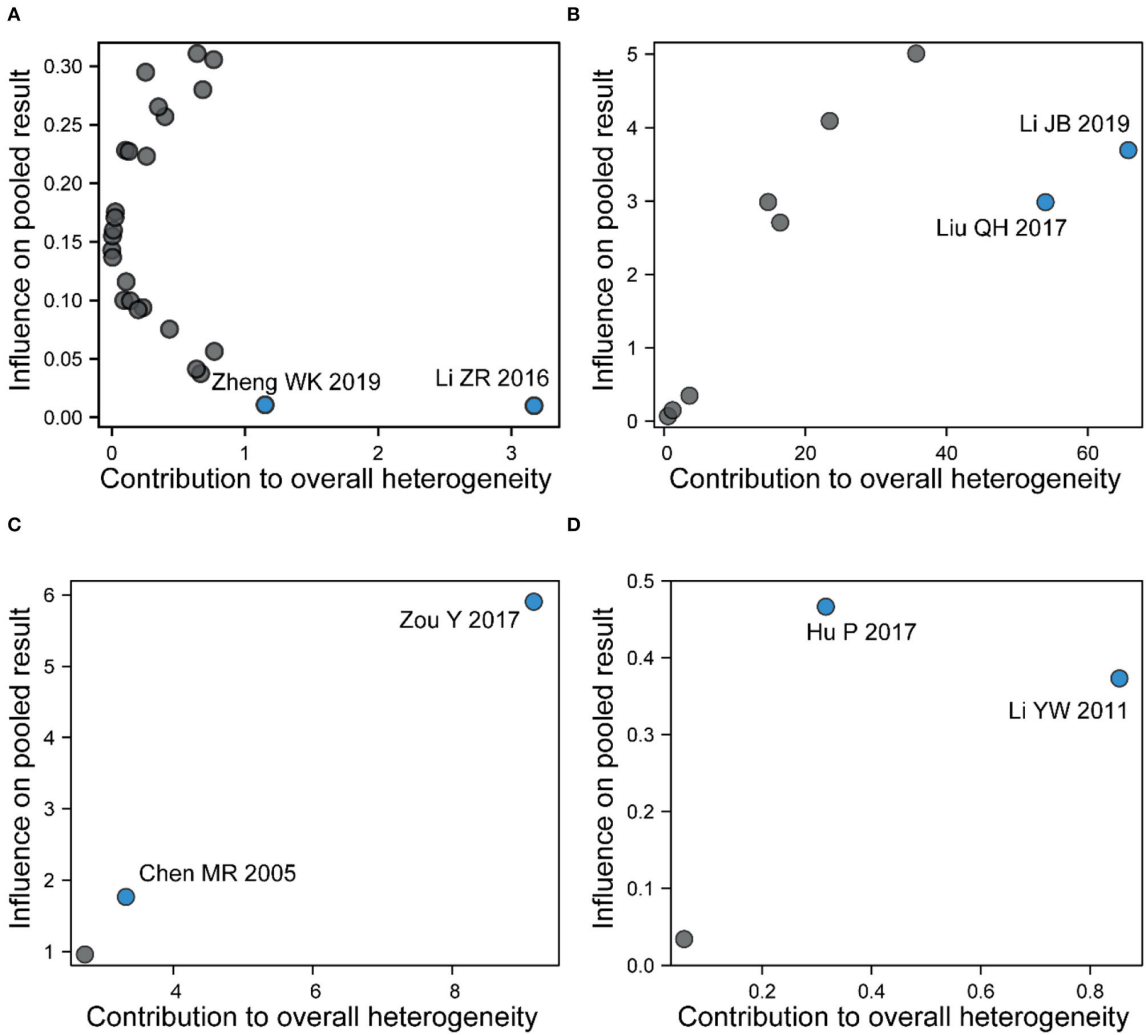
A Baujat plot for **(A)** total effective rate, **(B)** pain intensity, **(C)** pain threshold, and **(D)** recurrence rate. Each circle indicates an individual study, while the circle in blue indicates the study contributing more to heterogeneity and pooled effect.

As regards the high heterogeneity found in the comparison of acupuncture vs. MT on VAS pain score (I^2^ = 96%) and pain threshold (I^2^ = 69%), we performed the sensitivity analysis. By excluding studies individually, there was no significant change in the pooled effect size of the VAS score, but an extremely weak decrease in heterogeneity was observed when one study was excluded (Li et al., 2019) (Supplementary Table 5). Moreover, from the results of the Baujat plot, we found that two studies (Liu, 2017; Li et al., 2019) unduly influenced heterogeneity as well as the pooled effect of the VAS score (Figure 7). In the sensitivity analysis of pain threshold, the results showed that the I^2^ value significantly decreased from 69 to 0% after the exclusion of one study (Zou, 2017) (Supplementary Table 5), and there were two studies (Chen et al., 2005; Zou, 2017) that contributed overly to the heterogeneity from the results of the Baujat plot (Figure 7).

### Publication bias

**Figure 8:**
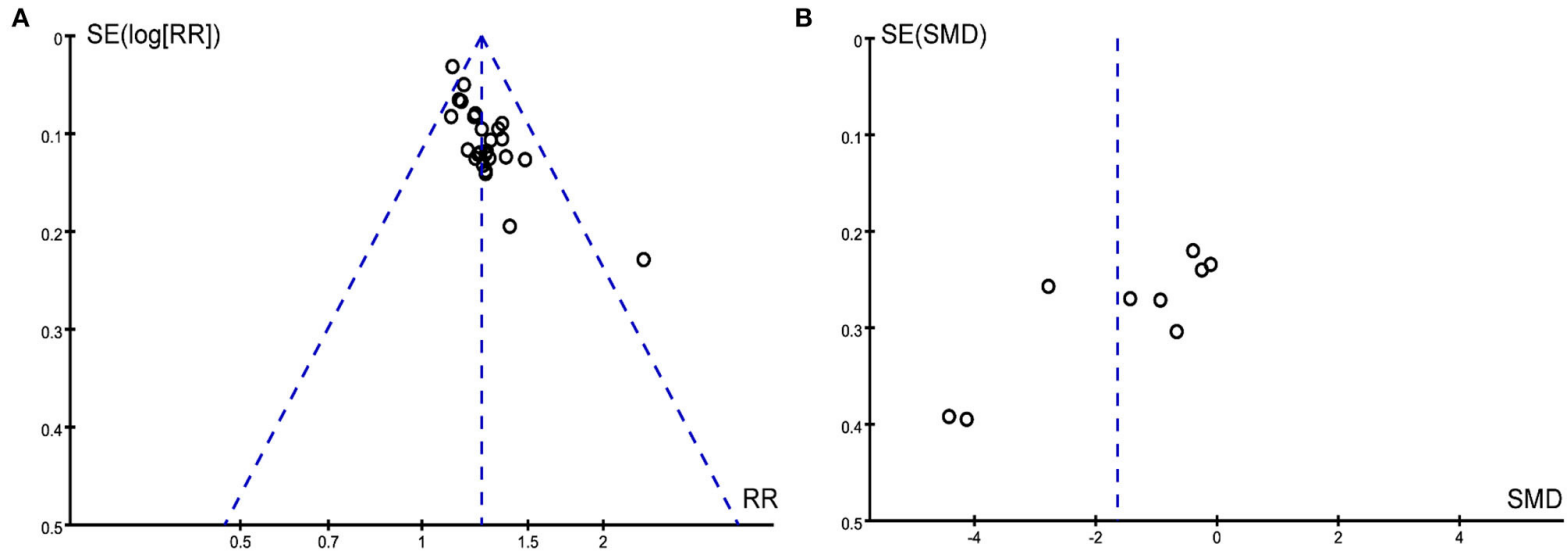
Funnel plots for **(A)** the total effective rate and **(B)** pain intensity.

We drew the funnel plot (Figure 8A) and used Peters' test (t = 1.500, *P* = 0.146) to calculate the outcome of the total effective rate, which indicated no publication bias. However, publication bias in the outcome of pain intensity may exist due to the asymmetrical funnel distribution and Egger's test (t = −3.562, *P* = 0.009) (Figure 8B).

### Certainty of evidence

The results of the GRADE score are summarized in Supplementary Table 6. The quality of evidence for these two outcomes (total effective rate and adverse events) was both rated as “moderate quality,” while the evidence of recurrence rate was rated as “low quality” and the rest of the outcomes (pain threshold and pain Intensity) were rated as “very low quality”.

## Discussion

This systematic review and meta-analysis demonstrated that acupuncture was more effective than MT or SA, with respect to reducing the VAS score, the recurrence rate and improving the total effective rate and pain threshold. In addition, a few adverse events were observed in the follow-up duration. Consistently, pooled effects of primary outcomes remained stable in the subgroup analysis apart from high heterogeneity in some results. Owing to concerns on the methodological quality and poor reporting quality, the aforementioned conclusions should be interpreted with great caution.

Our results showed that the quality of evidence on the outcomes ranged from very low to moderate. Moderate-certainty evidence showed that acupuncture was superior to MT in terms of the total effective rate and adverse events. However, we found that there was very low-certainty evidence showing that acupuncture offered greater pain relief than MT by reducing the VAS score and increasing the pain threshold, which was mainly related to the weakness of the study design and methodology in the included studies. Moreover, there was low-certainty evidence showing that a lower recurrence rate was observed in the patients with acupuncture treatment compared with MT at a long-term follow-up period.

Subgroup analysis of the pooled data was conducted to explore further the potential sources of significant heterogeneity observed in the 30 included studies. The results of the subgroup analysis showed that all types of acupuncture interventions obtained better results than MT, while the MA contributes to high heterogeneity in the outcome of pain intensity. Conversely, the EA subgroup significantly reduced the heterogeneity. As a type of acupuncture method, EA is being gradually used in clinical practice with unique advantages of combining traditional acupuncture therapy and absorbing the modern electronic theory. Compared with MA, EA was advocated to be more precise in the amount of needle stimulation. Furthermore, the SA group setting was an ideal method for controlling the placebo effects. Among the included studies, the SA group setting was used in two studies (Huang et al., 2019; Li et al., 2021). Huang and colleagues (Huang et al., 2019) found that acupuncture had a better effect than SA in relieving the symptoms of sciatic pain, and the same conclusion has been drawn in another study (Li et al., 2021). Unfortunately, no significant pooled effect was observed in the subgroup analysis of pain intensity, partly due to the limited number of included studies. So, it was necessary to investigate further the potential placebo effect in the future. Additionally, we observed that more sessions of acupuncture might show a certain degree of heterogeneity, which was most likely due to more reporting bias and difficulty in compliance in a long course of treatment.

Sensitivity analysis and Baujat plot were applied to evaluate the heterogeneity among studies in this review. Greater heterogeneity was observed in two studies on the total effective rate (Li and Kang, 2016; Zheng, 2019), while two other studies focused on pain intensity (Liu, 2017; Li et al., 2019). All the aforementioned studies belonged to the MA subgroup, and a few details on acupuncture were reported in two studies (Li et al., 2019; Zheng, 2019), which resulted in high heterogeneity in clinical methodologies. In addition, we focused on publication bias in primary outcomes. Significant publication bias was detected in pain intensity instead of the total effective rate. All the included studies in this meta-analysis were conducted in China, which was a source of potential publication bias.

In the theory of traditional Chinese medicine (TCM), sciatica belongs to the category of “bi” disease and “waist and leg pain” syndrome, which is mainly caused by the poor operation of “qi and blood” that flows through the bladder meridian and the gallbladder meridian. Acupuncture was suggested as a widely used non-pharmacological intervention for pain control, with the advantages of various treatment modalities (i.e., MA, EA, and WA) and minor side effects (Qiao et al., 2020). Inflammatory and neuropathic pain can be relieved effectively by acupuncture. It is the main mechanism involved in the alternation of blood rheology, immune defense, and neuromediators. Considering the particularity of acupuncture treatment, it is significant to assess the quality of the report on acupuncture intervention using the STRICTA checklist. The overall quality of interventions reported in the controlled trials of acupuncture was relatively good, though it still needs to be improved in details pertaining to needle insertion and treatment context. Additionally, we found that GB30, BL25, BL4, BL60, BL23, BL54, and GB34 were the most frequently used acupoints during acupuncture therapy. According to the TCM-based acupuncture meridian system, these selected primary acupoints are sciatic nerve-related acupoints consistent with clinically recommended commonly used primary acupoints (Zhang et al., 2020). With the more detailed elucidation of the stimulation mechanism of each acupoint, it is expected that more effective treatment strategies could be established based on the main symptoms of patients with sciatica.

Previous reviews investigated the effectiveness of acupuncture therapy for sciatica in 2015, while the lack of evaluation of acupuncture intervention details and evidence of quality, insufficient sample size, and a relatively inadequate assessment for heterogeneity limited the strength of conclusions (Ji et al., 2015; Qin et al., 2015). Compared to previous studies, our study had four novel advancements. First, more studies with a larger sample size were included to further enhance the reliability and stability of the meta-analysis. Second, the STRICTA checklist was added to raise the quality of reporting of the clinical trials of acupuncture. Next, acupuncture-associated subgroups not mentioned before (i.e., types of acupuncture interventions and sessions of treatment) were introduced for further analysis. In addition, the recurrence rate as a long-term outcome measure was considered as the secondary outcome mainly due to the characteristic of chronic and easy-to-relapse nature during the course of the disease. Finally, we assessed the quality level of the evidence and took into account the level of certainty of evidence for each outcome.

However, of course, there are still the following limitations in this review: (1) there were insufficient studies that compared acupuncture with SA supporting to avoid placebo effects, while fewer studies have been included currently; (2) the diversity of acupuncture methods, especially MA, contributed to the heterogeneity of the clinical outcome, and the results based on the STRICTA checklist found that the reporting of acupuncture details in existing studies is still incomplete, which limits our possibility to improve the quality of clinical evidence; and (3) the included studies still used efficiency as the primary assessment of acupuncture effectiveness. Nevertheless, pain intensity and pain threshold, indicators of patient pain evaluation, are still rarely used as the primary assessment in the literature. Therefore, changes in pain on patients with sciatica require further attention in the future.

In the future research on RCTs, well-designed and methodologically rigorous studies are needed to evaluate the true effects of acupuncture objectively on sciatica with a view to ultimately providing high-quality evidence for clinical practice. Fewer studies are currently undergoing pre-registration, and we strongly urge registry centers to prospectively register study protocols so that others follow these studies. In addition, it is quite difficult to achieve the blinding of acupuncturists but may be necessary and feasible for patients and outcome assessors. The assessment of outcome indicators also needs to be conducted on a uniform scale. In addition, high heterogeneity was reflected in a set of acupuncture-related factors, including acupoints, retention time of needles, acupuncturists' qualifications, and so on, and exploring heterogeneity in depth depends on the detailed description of the aforementioned factors. Therefore, we also expect that, with the help of the STRICTA checklist, more standardized acupuncture RCTs can be expected in the recent future.

## Conclusion

In summary, acupuncture therapy on sciatica was superior to MT or SA intervention, both in terms of clinical efficacy and safety, which suggested that acupuncture could be recommended as a feasible alternative therapy for patients with sciatica. However, given the high heterogeneity and low methodological quality of previous studies, future RCTs should be well-designed according to the rigorous methodology.

## Data availability statement

The datasets presented in this study can be found in online repositories. The names of the repository/repositories and accession number(s) can be found in the article/Supplementary material.

## Author contributions

GN, XinZ, and XiaZ designed the study. XinZ and XiaZ designed the search strategy. PH and ZH searched, screened studies, and assessed the risk of bias. ZZ and TH extracted the data. MY and ZH finished the reports on acupuncture interventions based on the STRICTA checklist. ZZ, TH, and PH analyzed the data. ZZ, XinZ, and YX wrote and drafted the manuscript. GN and XinZ provided administrative, technical, or administrative support. All authors read and approved the final manuscript.
