# Supplementary material for: The efficacy and safety of acupuncture therapy for sciatica: A systematic review and meta-analysis of randomized controlled trails
Source: Front Neurosci. 2023 Feb 9;17:1097830. doi: 10.3389/fnins.2023.1097830 (PMC9948020; doi:10.3389/fnins.2023.1097830)
Supplement: Supplementary file 2 [file Table_2.docx]

**Supplementary Table 2** Literature search strategy for this meta-analysis.

Table 2-1. Search strategy for Pubmed (52)

| **Number** | **Search terms** |
| --- | --- |
| #1 | Sciatica[Title/Abstract] OR sciatic neuralgia[Title/Abstract] OR sciatic neuropathy[Title/Abstract] |
| #2 | Acupuncture[Title/Abstract] OR electro-acupuncture [Title/Abstract] OR needle[Title/Abstract] OR needling[Title/Abstract] |
| #3 | #1 AND #2 |

Table 2-2. Search strategy for Web of Science (81)

| **Number** | **Search terms** |
| --- | --- |
| #1 | Sciatica(Topic)OR sciatic neuralgia(Topic)OR sciatic neuropathy(Topic) |
| #2 | Acupuncture(Topic)OR electro-acupuncture(Topic)OR needle(Topic)OR needling(Topic) |
| #3 | #1 AND #2 |

Table 2-3. Search strategy for Embase (240)

| **Number** | **Search terms** |
| --- | --- |
| #1 | ‘Sciatica’:ab,ti OR ‘sciatic neuralgia’:ab,ti OR ‘sciatic neuropathy’:ab,ti |
| #2 | ‘Acupuncture’:ab,ti OR ‘electro-acupuncture’:ab,ti OR ‘needle’:ab,ti OR ‘needling’:ab,ti |
| #3 | #1 AND #2 |

Table 2-4. Search strategy for the Cochrane Library (276)

| **Number** | **Search terms** |
| --- | --- |
| #1 | (Sciatica):ab,ti OR (sciatic neuralgia):ab,ti OR (sciatic neuropathy):ab,ti |
| #2 | (Acupuncture):ab,ti OR (electro-acupuncture):ab,ti OR (needle):ab,ti OR (needling):ab,ti |
| #3 | #1 AND #2 |

Table 2-5. Search strategy for CNKI database (807)

| **Number** | **Search terms** |
| --- | --- |
| #1 | (主题：坐骨神经痛)OR(主题：坐骨神经病)OR(主题：坐骨神经炎) |
| #2 | (主题：针刺)OR(主题：针灸)OR(主题：毫针)OR(主题：电针)OR(主题：温针)OR(主题：火针) |
| #3 | #1 AND #2 |

Table 2-6. Search strategy for VIP database (608)

| **Number** | **Search terms** |
| --- | --- |
| #1 | M=(坐骨神经痛OR坐骨神经病OR坐骨神经炎) |
| #2 | M=(针刺OR针灸OR毫针OR电针OR温针OR火针) |
| #3 | #1 AND #2 |

Table 2-7. Search strategy for Wang Fang database (700)

| **Number** | **Search terms** |
| --- | --- |
| #1 | 题名或关键词：(坐骨神经痛)OR题名或关键词：(坐骨神经病)OR题名或关键词：(坐骨神经炎) |
| #2 | 题名或关键词：(针刺)OR题名或关键词：(针灸)OR题名或关键词：(毫针)OR题名或关键词：(电针)OR题名或关键词：(温针)OR题名或关键词：(火针) |
| #3 | #1 AND #2 |
