# Supplementary material for: The efficacy and safety of acupuncture therapy for sciatica: A systematic review and meta-analysis of randomized controlled trails
Source: Front Neurosci. 2023 Feb 9;17:1097830. doi: 10.3389/fnins.2023.1097830 (PMC9948020; doi:10.3389/fnins.2023.1097830)
Supplement: Supplementary file 3 [file Table_3.docx]

**Supplementary Table 3** The assessment of risk of bias

**Huo F 2020**

| **Bias** | **Authors’ judgement** | **Support for judgement** |
| --- | --- | --- |
| Random sequence generation (selection bias) | Low risk | Using a random number table |
| Allocation concealment (selection bias) | Unclear risk | Not mentioned |
| Blinding of participants and personnel (performance bias) | High risk | No possible in the study. Acupuncturist selected appropriate acupoints for treatment according to the assessment of patients’ symptoms and feedback from participants. There was no method to ensure participants blinded. |
| Blinding of outcome assessment (detection bias) | Unclear risk | Not mentioned |
| Incomplete outcome data (attrition bias) | Low risk | No missing outcome data |
| Selective reporting (reporting bias) | Unclear risk | Results of secondary outcomes (pain threshold, recurrence rates, adverse events) were not reported. |
| Other bias | Low risk | No other sources of bias |

**Gu Y 2020**

| **Bias** | **Authors’ judgement** | **Support for judgement** |
| --- | --- | --- |
| Random sequence generation (selection bias) | Low risk | Using a random number table |
| Allocation concealment (selection bias) | Unclear risk | Not mentioned |
| Blinding of participants and personnel (performance bias) | High risk | No possible in the study |
| Blinding of outcome assessment (detection bias) | Unclear risk | Not mentioned |
| Incomplete outcome data (attrition bias) | Low risk | No missing outcome data |
| Selective reporting (reporting bias) | Unclear risk | Results of secondary outcomes (pain threshold, recurrence rates, adverse events) were not reported. |
| Other bias | Low risk | No other sources of bias |

**Zheng WK 2019**

| **Bias** | **Authors’ judgement** | **Support for judgement** |
| --- | --- | --- |
| Random sequence generation (selection bias) | Unclear risk | The specific method of randomization which was chosen was not reported. |
| Allocation concealment (selection bias) | Unclear risk | Not mentioned |
| Blinding of participants and personnel (performance bias) | High risk | No possible in the study |
| Blinding of outcome assessment (detection bias) | Unclear risk | Not mentioned |
| Incomplete outcome data (attrition bias) | Low risk | No missing outcome data |
| Selective reporting (reporting bias) | High risk | Not all the study’s primary outcomes have been reported. Only the total effective rate was reported. |
| Other bias | Low risk | No other sources of bias |

**Li JB 2019**

| **Bias** | **Authors’ judgement** | **Support for judgement** |
| --- | --- | --- |
| Random sequence generation (selection bias) | Unclear risk | The specific method of randomization which was chosen was not reported. (Details not reported) |
| Allocation concealment (selection bias) | Unclear risk | Not mentioned |
| Blinding of participants and personnel (performance bias) | High risk | No possible in the study |
| Blinding of outcome assessment (detection bias) | Unclear risk | Not mentioned |
| Incomplete outcome data (attrition bias) | Low risk | No missing outcome data |
| Selective reporting (reporting bias) | Unclear risk | Results of secondary outcomes (pain threshold, recurrence rates, adverse events) were not reported. |
| Other bias | Low risk | No other sources of bias |

**Jiang YQ 2018**

| **Bias** | **Authors’ judgement** | **Support for judgement** |
| --- | --- | --- |
| Random sequence generation (selection bias) | High risk | Sequence generated was based on date of admission |
| Allocation concealment (selection bias) | Unclear risk | Not mentioned |
| Blinding of participants and personnel (performance bias) | High risk | No possible in the study |
| Blinding of outcome assessment (detection bias) | Unclear risk | Not mentioned |
| Incomplete outcome data (attrition bias) | Low risk | No missing outcome data |
| Selective reporting (reporting bias) | High risk | Not all the study’s primary outcomes have been reported. Only the total effective rate was reported. |
| Other bias | Low risk | No other sources of bias |

**Zou Y 2017**

| **Bias** | **Authors’ judgement** | **Support for judgement** |
| --- | --- | --- |
| Random sequence generation (selection bias) | Unclear risk | Details not reported |
| Allocation concealment (selection bias) | Unclear risk | Not mentioned |
| Blinding of participants and personnel (performance bias) | High risk | No possible in the study |
| Blinding of outcome assessment (detection bias) | Unclear risk | Not mentioned |
| Incomplete outcome data (attrition bias) | Low risk | No missing outcome data |
| Selective reporting (reporting bias) | Unclear risk | Results of secondary outcomes (pain threshold, recurrence rates, adverse events) were not reported. |
| Other bias | Low risk | No other sources of bias |

**Yu HW 2017**

| **Bias** | **Authors’ judgement** | **Support for judgement** |
| --- | --- | --- |
| Random sequence generation (selection bias) | Low risk | Using a random number table |
| Allocation concealment (selection bias) | Unclear risk | Not mentioned |
| Blinding of participants and personnel (performance bias) | High risk | No possible in the study |
| Blinding of outcome assessment (detection bias) | Unclear risk | Not mentioned |
| Incomplete outcome data (attrition bias) | Low risk | No missing outcome data |
| Selective reporting (reporting bias) | High risk | Not all the study’s primary outcomes have been reported. Only the total effective rate was reported. |
| Other bias | Low risk | No other sources of bias |

**Liu JY 2015**

| **Bias** | **Authors’ judgement** | **Support for judgement** |
| --- | --- | --- |
| Random sequence generation (selection bias) | Unclear risk | Details not reported |
| Allocation concealment (selection bias) | Unclear risk | Not mentioned |
| Blinding of participants and personnel (performance bias) | High risk | No possible in the study |
| Blinding of outcome assessment (detection bias) | Unclear risk | Not mentioned |
| Incomplete outcome data (attrition bias) | Low risk | No missing outcome data |
| Selective reporting (reporting bias) | High risk | Not all the study’s primary outcomes have been reported. Only the total effective rate was reported. |
| Other bias | Low risk | No other sources of bias |

**Shang HM 2014**

| **Bias** | **Authors’ judgement** | **Support for judgement** |
| --- | --- | --- |
| Random sequence generation (selection bias) | Unclear risk | Details not reported |
| Allocation concealment (selection bias) | Unclear risk | Not mentioned |
| Blinding of participants and personnel (performance bias) | High risk | No possible in the study |
| Blinding of outcome assessment (detection bias) | Unclear risk | Not mentioned |
| Incomplete outcome data (attrition bias) | Low risk | Wrong data have been recalculated using appropriate methods. |
| Selective reporting (reporting bias) | High risk | Not all the study’s primary outcomes have been reported. Only the total effective rate was reported. |
| Other bias | Low risk | No other sources of bias |

**Liu QH 2017**

| **Bias** | **Authors’ judgement** | **Support for judgement** |
| --- | --- | --- |
| Random sequence generation (selection bias) | Low risk | Using a random number table |
| Allocation concealment (selection bias) | Unclear risk | Not mentioned |
| Blinding of participants and personnel (performance bias) | High risk | No possible in the study |
| Blinding of outcome assessment (detection bias) | Unclear risk | Not mentioned |
| Incomplete outcome data (attrition bias) | Low risk | No missing outcome data |
| Selective reporting (reporting bias) | Unclear risk | All the primary outcomes were reported, but the secondary outcomes (pain threshold, recurrence rates, adverse events) were not reported. |
| Other bias | Low risk | No other sources of bias |

**Zeng Y 2012**

| **Bias** | **Authors’ judgement** | **Support for judgement** |
| --- | --- | --- |
| Random sequence generation (selection bias) | Unclear risk | Details not reported |
| Allocation concealment (selection bias) | Unclear risk | Not mentioned |
| Blinding of participants and personnel (performance bias) | High risk | No possible in the study |
| Blinding of outcome assessment (detection bias) | Unclear risk | Not mentioned |
| Incomplete outcome data (attrition bias) | Low risk | No missing outcome data |
| Selective reporting (reporting bias) | High risk | Not all of the study’s primary outcomes have been reported. Only the total effective rate was reported. |
| Other bias | Low risk | No other sources of bias |

**Zhang Z 2012**

| **Bias** | **Authors’ judgement** | **Support for judgement** |
| --- | --- | --- |
| Random sequence generation (selection bias) | Unclear risk | Details not reported |
| Allocation concealment (selection bias) | Unclear risk | Not mentioned |
| Blinding of participants and personnel (performance bias) | High risk | No possible in the study |
| Blinding of outcome assessment (detection bias) | Unclear risk | Not mentioned |
| Incomplete outcome data (attrition bias) | Low risk | Missing data have been imputed using appropriate methods. |
| Selective reporting (reporting bias) | High risk | Not all of the study’s primary outcomes have been reported. Only the total effective rate and adverse events were reported. |
| Other bias | Low risk | No other sources of bias |

**Wang JM 2020**

| **Bias** | **Authors’ judgement** | **Support for judgement** |
| --- | --- | --- |
| Random sequence generation (selection bias) | Low risk | Using a computer random number generator |
| Allocation concealment (selection bias) | Unclear risk | Not mentioned |
| Blinding of participants and personnel (performance bias) | High risk | No possible in the study |
| Blinding of outcome assessment (detection bias) | Unclear risk | Not mentioned |
| Incomplete outcome data (attrition bias) | Low risk | No missing outcome data |
| Selective reporting (reporting bias) | High risk | Only the total effective rate and adverse events were reported. |
| Other bias | Low risk | No other sources of bias |

**Li JY 2018**

| **Bias** | **Authors’ judgement** | **Support for judgement** |
| --- | --- | --- |
| Random sequence generation (selection bias) | Low risk | Table of random numbers was used. |
| Allocation concealment (selection bias) | Unclear risk | Not mentioned |
| Blinding of participants and personnel (performance bias) | High risk | No possible in the study |
| Blinding of outcome assessment (detection bias) | Unclear risk | Not mentioned |
| Incomplete outcome data (attrition bias) | Low risk | No missing outcome data |
| Selective reporting (reporting bias) | High risk | Only the total effective rate was reported. |
| Other bias | Low risk | No other sources of bias |

**Wang CJ 2016**

| **Bias** | **Authors’ judgement** | **Support for judgement** |
| --- | --- | --- |
| Random sequence generation (selection bias) | Unclear risk | Details not reported |
| Allocation concealment (selection bias) | Unclear risk | Not mentioned |
| Blinding of participants and personnel (performance bias) | High risk | No possible in the study |
| Blinding of outcome assessment (detection bias) | Unclear risk | Not mentioned |
| Incomplete outcome data (attrition bias) | High risk | The data of pain threshold was not reported. |
| Selective reporting (reporting bias) | High risk | Not all of the study’s primary outcomes have been reported. Only the total effective rate and pain threshold was reported. |
| Other bias | Low risk | No other sources of bias |

**Wang ZM 2017**

| **Bias** | **Authors’ judgement** | **Support for judgement** |
| --- | --- | --- |
| Random sequence generation (selection bias) | Unclear risk | Details not reported |
| Allocation concealment (selection bias) | Unclear risk | Not mentioned |
| Blinding of participants and personnel (performance bias) | High risk | No possible in the study |
| Blinding of outcome assessment (detection bias) | Unclear risk | Not mentioned |
| Incomplete outcome data (attrition bias) | Low risk | No missing outcome data |
| Selective reporting (reporting bias) | High risk | Only the total effective rate was reported. |
| Other bias | Low risk | No other sources of bias |

**Nie JD 2015**

| **Bias** | **Authors’ judgement** | **Support for judgement** |
| --- | --- | --- |
| Random sequence generation (selection bias) | Low risk | The trail was a randomized, parallel, controlled group study. |
| Allocation concealment (selection bias) | Unclear risk | Not mentioned |
| Blinding of participants and personnel (performance bias) | High risk | No possible in the study |
| Blinding of outcome assessment (detection bias) | Unclear risk | Not mentioned |
| Incomplete outcome data (attrition bias) | Low risk | No missing outcome data |
| Selective reporting (reporting bias) | High risk | Only the total effective rate was reported. |
| Other bias | Low risk | No other sources of bias |

**Jiang BY 2012**

| **Bias** | **Authors’ judgement** | **Support for judgement** |
| --- | --- | --- |
| Random sequence generation (selection bias) | Unclear risk | Details not reported |
| Allocation concealment (selection bias) | Unclear risk | Not mentioned |
| Blinding of participants and personnel (performance bias) | High risk | No possible in the study |
| Blinding of outcome assessment (detection bias) | Unclear risk | Not mentioned |
| Incomplete outcome data (attrition bias) | Low risk | Errors have been corrected using appropriate methods. |
| Selective reporting (reporting bias) | High risk | Only the total effective rate was reported. |
| Other bias | Low risk | No other sources of bias |

**Liu BL 2012**

| **Bias** | **Authors’ judgement** | **Support for judgement** |
| --- | --- | --- |
| Random sequence generation (selection bias) | Unclear risk | Details not reported |
| Allocation concealment (selection bias) | Unclear risk | Not mentioned |
| Blinding of participants and personnel (performance bias) | High risk | No possible in the study |
| Blinding of outcome assessment (detection bias) | Unclear risk | Not mentioned |
| Incomplete outcome data (attrition bias) | Low risk | No missing outcome data |
| Selective reporting (reporting bias) | High risk | Only the pain threshold was reported. |
| Other bias | Low risk | No other sources of bias |

**Li ZR 2016**

| **Bias** | **Authors’ judgement** | **Support for judgement** |
| --- | --- | --- |
| Random sequence generation (selection bias) | Unclear risk | Details not reported |
| Allocation concealment (selection bias) | Unclear risk | Not mentioned |
| Blinding of participants and personnel (performance bias) | High risk | No possible in the study |
| Blinding of outcome assessment (detection bias) | Unclear risk | Not mentioned |
| Incomplete outcome data (attrition bias) | Low risk | No missing outcome data |
| Selective reporting (reporting bias) | High risk | Only the total effective rate was reported. |
| Other bias | Low risk | No other sources of bias |

**Hu P 2017**

| **Bias** | **Authors’ judgement** | **Support for judgement** |
| --- | --- | --- |
| Random sequence generation (selection bias) | Unclear risk | Details not reported |
| Allocation concealment (selection bias) | Unclear risk | Not mentioned |
| Blinding of participants and personnel (performance bias) | High risk | No possible in the study |
| Blinding of outcome assessment (detection bias) | Unclear risk | Not mentioned |
| Incomplete outcome data (attrition bias) | Low risk | No missing outcome data |
| Selective reporting (reporting bias) | High risk | Only the total effective rate was reported. |
| Other bias | Low risk | No other sources of bias |

**Wei Q 2016**

| **Bias** | **Authors’ judgement** | **Support for judgement** |
| --- | --- | --- |
| Random sequence generation (selection bias) | High risk | Sequence was generated by the time of the admission order. |
| Allocation concealment (selection bias) | Unclear risk | Not mentioned |
| Blinding of participants and personnel (performance bias) | High risk | No possible in the study |
| Blinding of outcome assessment (detection bias) | Unclear risk | Not mentioned |
| Incomplete outcome data (attrition bias) | Low risk | No missing outcome data |
| Selective reporting (reporting bias) | High risk | Only the total effective rate and recurrence rates was reported. |
| Other bias | Low risk | No other sources of bias |

**Ai XJ 2015**

| **Bias** | **Authors’ judgement** | **Support for judgement** |
| --- | --- | --- |
| Random sequence generation (selection bias) | Low risk | The trail was a randomized, parallel, controlled group study. |
| Allocation concealment (selection bias) | Unclear risk | Not mentioned |
| Blinding of participants and personnel (performance bias) | High risk | No possible in the study |
| Blinding of outcome assessment (detection bias) | Unclear risk | Not mentioned |
| Incomplete outcome data (attrition bias) | Low risk | No missing outcome data |
| Selective reporting (reporting bias) | High risk | Only the total effective rate and adverse events was reported. |
| Other bias | Low risk | No other sources of bias |

**Zhai LH 2012**

| **Bias** | **Authors’ judgement** | **Support for judgement** |
| --- | --- | --- |
| Random sequence generation (selection bias) | Unclear risk | Details not reported |
| Allocation concealment (selection bias) | Unclear risk | Not mentioned |
| Blinding of participants and personnel (performance bias) | High risk | No possible in the study |
| Blinding of outcome assessment (detection bias) | Unclear risk | Not mentioned |
| Incomplete outcome data (attrition bias) | Low risk | No missing outcome data |
| Selective reporting (reporting bias) | High risk | Only the total effective rate was reported. |
| Other bias | Low risk | No other sources of bias |

**Chen MR 2005**

| **Bias** | **Authors’ judgement** | **Support for judgement** |
| --- | --- | --- |
| Random sequence generation (selection bias) | Low risk | Table of random number was used. |
| Allocation concealment (selection bias) | Unclear risk | Not mentioned |
| Blinding of participants and personnel (performance bias) | High risk | No possible in the study |
| Blinding of outcome assessment (detection bias) | Unclear risk | Not mentioned |
| Incomplete outcome data (attrition bias) | Low risk | No missing outcome data |
| Selective reporting (reporting bias) | High risk | Only the total effective rate and pain threshold was reported. |
| Other bias | Low risk | No other sources of bias |

**Huang JY 2015**

| **Bias** | **Authors’ judgement** | **Support for judgement** |
| --- | --- | --- |
| Random sequence generation (selection bias) | Unclear risk | Details not reported |
| Allocation concealment (selection bias) | Unclear risk | Not mentioned |
| Blinding of participants and personnel (performance bias) | High risk | No possible in the study |
| Blinding of outcome assessment (detection bias) | Unclear risk | Not mentioned |
| Incomplete outcome data (attrition bias) | Low risk | No missing outcome data |
| Selective reporting (reporting bias) | Unclear risk | All the primary outcomes were reported, but the secondary outcomes (pain threshold, recurrence rates, adverse events) were not reported. |
| Other bias | Low risk | No other sources of bias |

**Li YW 2011**

| **Bias** | **Authors’ judgement** | **Support for judgement** |
| --- | --- | --- |
| Random sequence generation (selection bias) | Unclear risk | Details not reported |
| Allocation concealment (selection bias) | Unclear risk | Not mentioned |
| Blinding of participants and personnel (performance bias) | High risk | Not blinded |
| Blinding of outcome assessment (detection bias) | Unclear risk | Not mentioned |
| Incomplete outcome data (attrition bias) | Low risk | No missing outcome data |
| Selective reporting (reporting bias) | Low risk | All outcomes in the protocol were reported |
| Other bias | Low risk | No other sources of bias |

**Ye XC 2015**

| **Bias** | **Authors’ judgement** | **Support for judgement** |
| --- | --- | --- |
| Random sequence generation (selection bias) | Low risk | The trail was a randomized, parallel, controlled group study. |
| Allocation concealment (selection bias) | Unclear risk | Not mentioned |
| Blinding of participants and personnel (performance bias) | High risk | Not blinded |
| Blinding of outcome assessment (detection bias) | Unclear risk | Not mentioned |
| Incomplete outcome data (attrition bias) | Low risk | No missing outcome data |
| Selective reporting (reporting bias) | High risk | Not all the study’s primary outcomes have been reported. |
| Other bias | Low risk | No other sources of bias |

**Huang ZL 2019**

| **Bias** | **Authors’ judgement** | **Support for judgement** |
| --- | --- | --- |
| Random sequence generation (selection bias) | Low risk | Computer software-generated random numbers |
| Allocation concealment (selection bias) | Low risk | Random numbers and group assigned were kept in sealed opaque envelopes. |
| Blinding of participants and personnel (performance bias) | Low risk | In order to ensure participants blinded, the acupuncture  group placed the same fixed adhesive pad as the sham  acupuncture group. |
| Blinding of outcome assessment (detection bias) | Low risk | Outcome assessors and statisticians were blinded to intervention assignment. |
| Incomplete outcome data (attrition bias) | Low risk | No missing outcome data |
| Selective reporting (reporting bias) | High risk | Only pain intensity was reported. |
| Other bias | Low risk | No other sources of bias |

**Li Cheng 2021**

| **Bias** | **Authors’ judgement** | **Support for judgement** |
| --- | --- | --- |
| Random sequence generation (selection bias) | Unclear risk | Details not reported |
| Allocation concealment (selection bias) | Unclear risk | Not mentioned |
| Blinding of participants and personnel (performance bias) | Low risk | In order to ensure participants blinded, the acupuncture  group placed the same fixed adhesive pad as the sham  acupuncture group. |
| Blinding of outcome assessment (detection bias) | Unclear risk | Not mentioned |
| Incomplete outcome data (attrition bias) | Low risk | No missing outcome data |
| Selective reporting (reporting bias) | High risk | Only pain intensity was reported. |
| Other bias | Low risk | No other sources of bias |
