# Supplementary material for: The efficacy and safety of acupuncture therapy for sciatica: A systematic review and meta-analysis of randomized controlled trails
Source: Front Neurosci. 2023 Feb 9;17:1097830. doi: 10.3389/fnins.2023.1097830 (PMC9948020; doi:10.3389/fnins.2023.1097830)
Supplement: Supplementary file 5 [file Table_5.docx]

**Supplementary Table 5** – Sensitivity analyses for outcomes.

**Total effective rate**

| **Excluded study** | **Intervention group(n)** | **Control group(n)** | **RR/SMD (95% CI)** | **P value** | **Heterogeneity test** | **Effect model** |
| --- | --- | --- | --- | --- | --- | --- |
| Before excluding | 1229 | 1203 | 1.25 [1.21, 1.30] | <0.001 | P = 0.20, I² = 19% | Fixed |
| Huo F 2020 | 1169 | 1143 | 1.26 [1.21, 1.31] | <0.001 | P = 0.17, I² = 22% | Fixed |
| Gu Y 2020 | 1194 | 1168 | 1.25 [1.21, 1.30] | <0.001 | P = 0.15, I² = 23% | Fixed |
| Zheng WK 2019 | 1074 | 1048 | 1.28 [1.22, 1.33] | <0.001 | P = 0.65, I² = 0% | Fixed |
| Li JB 2019 | 1183 | 1157 | 1.26 [1.21, 1.30] | <0.001 | P = 0.18, I² = 20% | Fixed |
| Jiang YQ 2018 | 1169 | 1143 | 1.25 [1.20, 1.29] | <0.001 | P = 0.23, I² = 17% | Fixed |
| Zou Y 2017 | 1199 | 1173 | 1.25 [1.21, 1.30] | <0.001 | P = 0.20, I² = 19% | Fixed |
| Yu HW 2017 | 1201 | 1181 | 1.25 [1.21, 1.30] | <0.001 | P = 0.16, I² = 22% | Fixed |
| Liu JY 2015 | 1181 | 1155 | 1.25 [1.20, 1.29] | <0.001 | P = 0.29, I² = 12% | Fixed |
| Shang HM 2014 | 1169 | 1143 | 1.25 [1.20, 1.29] | <0.001 | P = 0.21, I² = 18% | Fixed |
| Liu QH 2017 | 1187 | 1162 | 1.25 [1.21, 1.30] | <0.001 | P = 0.16, I² = 22% | Fixed |
| Zeng Y 2012 | 1164 | 1138 | 1.25 [1.21, 1.30] | <0.001 | P = 0.15, I² = 23% | Fixed |
| Zhang Z 2012 | 1154 | 1133 | 1.25 [1.20, 1.29] | <0.001 | P = 0.21, I² = 18% | Fixed |
| Wang JM 2020 | 1189 | 1163 | 1.25 [1.21, 1.30] | <0.001 | P = 0.16, I² = 22% | Fixed |
| Li JY 2018 | 1196 | 1170 | 1.25 [1.21, 1.30] | <0.001 | P = 0.16, I² = 22% | Fixed |
| Wang CJ 2016 | 1139 | 1113 | 1.26 [1.21, 1.31] | <0.001 | P = 0.17, I² = 21% | Fixed |
| Wang ZM 2017 | 1204 | 1178 | 1.25 [1.21, 1.30] | <0.001 | P = 0.16, I² = 22% | Fixed |
| Nie JD 2015 | 1190 | 1166 | 1.25 [1.21, 1.30] | <0.001 | P = 0.17, I² = 21% | Fixed |
| Jiang BY 2012 | 1188 | 1162 | 1.26 [1.21, 1.31] | <0.001 | P = 0.19, I² = 20% | Fixed |
| Li ZR 2016 | 1199 | 1173 | 1.24 [1.19, 1.28] | <0.001 | P = 0.67, I² = 0% | Fixed |
| Hu P 2017 | 1189 | 1163 | 1.25 [1.21, 1.30] | <0.001 | P = 0.17, I² = 21% | Fixed |
| Wei Q 2016 | 1214 | 1188 | 1.25 [1.21, 1.30] | <0.001 | P = 0.18, I² = 20% | Fixed |
| Ai XJ 2015 | 1199 | 1173 | 1.25 [1.21, 1.30] | <0.001 | P = 0.16, I² = 22% | Fixed |
| Zhai LH 2012 | 1201 | 1175 | 1.25 [1.21, 1.30] | <0.001 | P = 0.16, I² = 22% | Fixed |
| Chen MR 2005 | 1199 | 1173 | 1.25 [1.21, 1.30] | <0.001 | P = 0.16, I² = 22% | Fixed |
| Huang JY 2015 | 1194 | 1168 | 1.25 [1.21, 1.30] | <0.001 | P = 0.17, I² = 22% | Fixed |
| Li YW 2011 | 1180 | 1166 | 1.25 [1.21, 1.30] | <0.001 | P = 0.16, I² = 22% | Fixed |

**Pain intensity**

| **Excluded study** | **Intervention group(n)** | **Control group(n)** | **RR/SMD (95% CI)** | **P value** | **Heterogeneity test** | **Effect model** |
| --- | --- | --- | --- | --- | --- | --- |
| Before excluding | 358 | 343 | -1.65 [-2.57, -0.72] | <0.001 | P < 0.01, I² = 96% | Random |
| Huo F 2020 | 298 | 283 | -1.50 [-2.46, -0.55] | <0.001 | P < 0.01, I² = 96% | Random |
| Gu Y 2020 | 323 | 308 | -1.68 [-2.73, -0.62] | <0.001 | P < 0.01, I² = 97% | Random |
| Li JB 2019 | 312 | 297 | -1.31 [-2.14, -0.47] | <0.001 | P < 0.01, I² = 95% | Random |
| Liu QH 2017 | 316 | 302 | -1.34 [-2.21, -0.47] | <0.001 | P < 0.01, I² = 96% | Random |
| Huang JY 2015 | 323 | 308 | -1.82 [-2.85, -0.80] | <0.001 | P < 0.01, I² = 96% | Random |
| Li YW 2011 | 309 | 306 | -1.81 [-2.85, -0.77] | <0.001 | P < 0.01, I² = 96% | Random |
| Ye XC 2015 | 327 | 313 | -1.74 [-2.79, -0.69] | <0.001 | P < 0.01, I² = 97% | Random |
| Huang ZL 2019 | 335 | 320 | -1.77 [-2.80, -0.74] | <0.001 | P < 0.01, I² = 97% | Random |
| Cheng L 2021 | 321 | 307 | -1.84 [-2.85, -0.84] | <0.001 | P < 0.01, I² = 96% | Random |

**Pain threshold**

| **Excluded study** | **Intervention group(n)** | **Control group(n)** | **RR/SMD (95% CI)** | **P value** | **Heterogeneity test** | **Effect model** |
| --- | --- | --- | --- | --- | --- | --- |
| Before excluding | 90 | 80 | 2.07 [1.38, 2.75] | <0.001 | P = 0.04, I² = 69% | Random |
| Zou Y 2017 | 60 | 50 | 2.42 [1.92, 2.92] | <0.001 | P = 0.53, I² = 0% | Fix |
| Liu BL 2012 | 60 | 60 | 2.00 [0.92, 3.09] | <0.001 | P = 0.02, I² = 83% | Random |
| Chen MR 2005 | 60 | 50 | 1.82 [1.06, 2.59] | <0.001 | P = 0.10, I² = 64% | Random |

**Recurrence rate**

| **Excluded study** | **Intervention group(n)** | **Control group(n)** | **RR/SMD (95% CI)** | **P value** | **Heterogeneity test** | **Effect model** |
| --- | --- | --- | --- | --- | --- | --- |
| Before excluding | 104 | 92 | 0.27 [0.13, 0.56] | <0.001 | P = 0.49, I² = 0% | Fix |
| Hu P 2017 | 64 | 52 | 0.18 [0.05, 0.57] | <0.001 | P = 0.50, I² = 0% | Fix |
| Wei Q 2016 | 89 | 77 | 0.26 [0.12, 0.57] | <0.001 | P = 0.23, I² = 29% | Fix |
| Li YW 2011 | 55 | 55 | 0.38 [0.16, 0.88] | 0.020 | P = 0.90, I² = 0% | Fix |
