# Supplementary material for: The efficacy and safety of acupuncture therapy for sciatica: A systematic review and meta-analysis of randomized controlled trails
Source: Front Neurosci. 2023 Feb 9;17:1097830. doi: 10.3389/fnins.2023.1097830 (PMC9948020; doi:10.3389/fnins.2023.1097830)
Supplement: Supplementary file 6 [file Table_6.docx]

**Supplementary Table 6** The quality of evidence for GRADE score.

| **Certainty assessment** | | | | | | | **No. of patients** | | **Effect** | | **Certainty** |
| --- | --- | --- | --- | --- | --- | --- | --- | --- | --- | --- | --- |
| **№ of studies** | **Study design** | **Risk of bias** | **Inconsistency** | **Indirectness** | **Imprecision** | **Other considerations** | **Acupuncture** | **Control** | **Relative (95% CI)** | **Absolute (95% CI)** |  |
| **Total Effective Rate** | | | | | | | | | | | |
| 26 | randomised trials | serious^a^ | not serious | not serious | not serious | none | 1147/1229 (93.3%) | 897/1203 (74.6%) | RR 1.25 (1.21 to 1.30) | 186 more per 1,000 (from 157 more to 224 more) | ⨁⨁⨁◯ Moderate |
| **Pain threshold** | | | | | | | | | | | |
| 3 | randomised trials | serious^a^ | serious^b^ | not serious | serious^c^ | none | 90 | 80 | - | SMD 2.07 higher (1.38 higher to 2.75 higher) | ⨁◯◯◯ Very low |
| **Recurrence rate** | | | | | | | | | | | |
| 3 | randomised trials | serious^a^ | not serious | not serious | serious^c^ | none | 8/104 (7.7%) | 27/92 (29.3%) | RR 0.27 (0.13 to 0.56) | 214 fewer per 1,000 (from 255 fewer to 129 fewer) | ⨁⨁◯◯ Low |
| **Pain Intensity** | | | | | | | | | | | |
| 9 | randomised trials | serious^a^ | serious^b^ | not serious | not serious | publication bias strongly suspected^d^ | 358 | 343 | - | SMD 1.75 lower (1.86 lower to 1.63 lower) | ⨁◯◯◯ Very low |
| **Adverse events** | | | | | | | | | | | |
| 5 | randomised trials | serious^a^ | not serious | not serious | not serious | none | 10/200  (5.0%) | 29/199 (14.6%) | RR 0.38 (0.19 to 0.72) | 90 fewer per 1,000 (from 118 fewer to 41 fewer) | ⨁⨁⨁◯ Moderate |

**Notes**: CI: confidence interval; RR: risk ratio; SMD: standardised mean difference

**Explanations**

a. >50% of studies with high risk of bias

b. I^2^ is >50%

c. total sample size is <200

d. tested by Egger’s test
