# Supplementary material for: The efficacy and safety of acupuncture therapy for sciatica: A systematic review and meta-analysis of randomized controlled trails
Source: Front Neurosci. 2023 Feb 9;17:1097830. doi: 10.3389/fnins.2023.1097830 (PMC9948020; doi:10.3389/fnins.2023.1097830)
Supplement: Supplementary file 7 [file Data_Sheet_1.docx]

**Supplementary Figure**

**
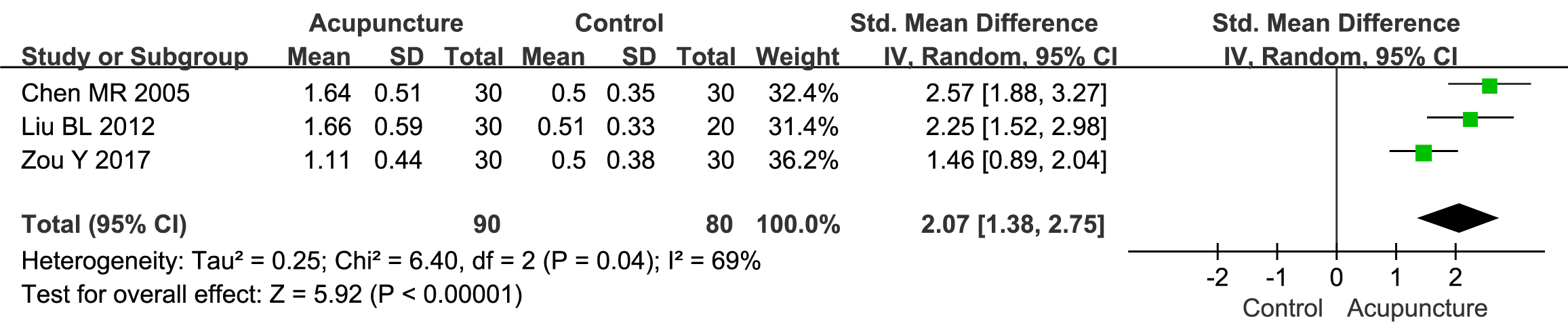
**

**Supplementary Figure 1** Forest plot for pain threshold of acupuncture versus medicine treatment


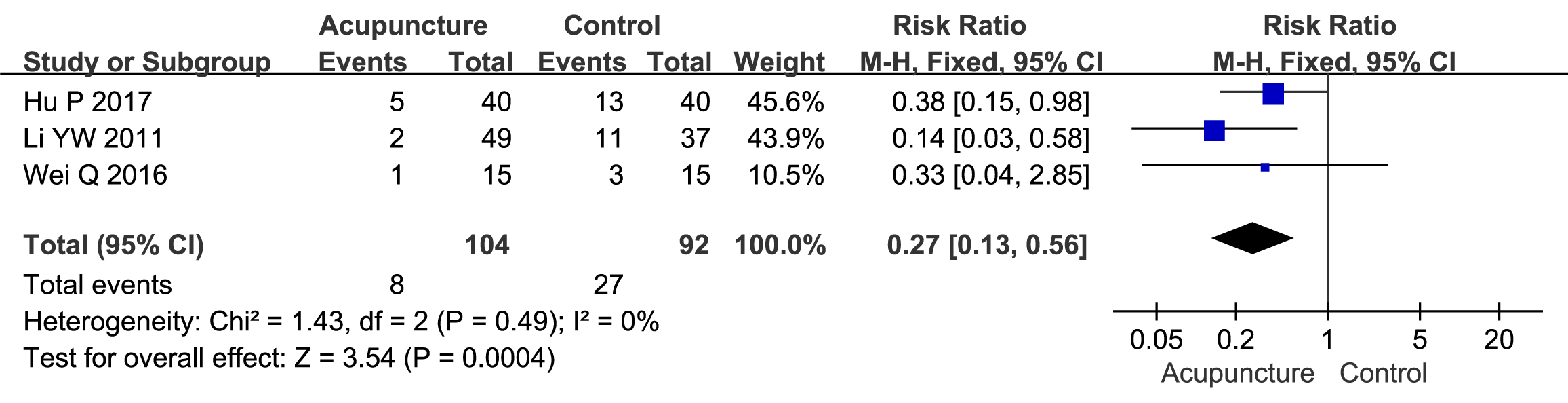


**Supplementary Figure 2** Forest plot for recurrence rate of acupuncture versus medicine treatment


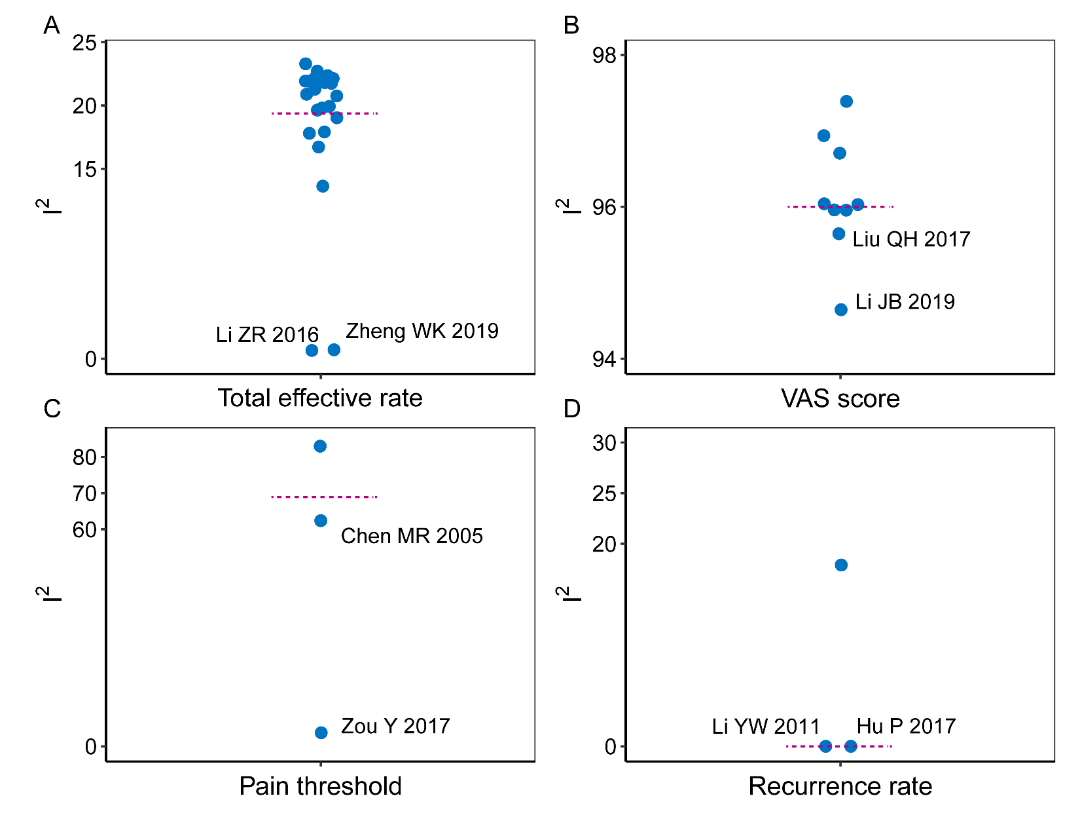


**Supplementary Figure 3** (A) total effective rate (B) pain Intensity (C) pain threshold (D) recurrence rate of sensitive analysis. The purple line indicates the heterogeneity value (I^2^) before excluded, the blue points indicate I^2^ after excluded one study.
